# Purification and characterisation of dsRNA using ion pair reverse phase chromatography and mass spectrometry

**DOI:** 10.1016/j.chroma.2016.12.062

**Published:** 2017-02-10

**Authors:** Alison O. Nwokeoji, An-Wen Kung, Peter M. Kilby, David E. Portwood, Mark J. Dickman

**Affiliations:** a Department of Chemical and Biological Engineering, ChELSI Institute, Mappin Street, University of Sheffield, S1 3JD, UK; b Syngenta, Jealott's Hill International Research Centre, Bracknell, Berkshire, RG42 6EY, UK

**Keywords:** Double stranded RNA purification, RNase mass mapping, Ion pair reverse phase HPLC, Mass spectrometry

## Abstract

RNA interference has provided valuable insight into a wide range of biological systems and is a powerful tool for the analysis of gene function. The exploitation of this pathway to block the expression of specific gene targets holds considerable promise for the development of novel RNAi-based insect management strategies. In addition, there are a wide number of future potential applications of RNAi to control agricultural insect pests as well as its use for prevention of diseases in beneficial insects. The potential to synthesise large quantities of dsRNA by *in-vitro* transcription or in bacterial systems for RNA interference applications has generated significant demand for the development and application of high throughput analytical tools for the rapid extraction, purification and analysis of dsRNA. Here we have developed analytical methods that enable the rapid purification of dsRNA from associated impurities from bacterial cells in conjunction with downstream analyses. We have optimised TRIzol extractions in conjunction with a single step protocol to remove contaminating DNA and ssRNA, using RNase T1/DNase I digestion under high-salt conditions in combination with solid phase extraction to purify the dsRNA. In addition, we have utilised and developed IP RP HPLC for the rapid, high resolution analysis of the dsRNA. Furthermore, we have optimised base-specific cleavage of dsRNA by RNase A and developed a novel method utilising RNase T1 for RNase mass mapping approaches to further characterise the dsRNA using liquid chromatography interfaced with mass spectrometry.

## Highlights

• rapid purification of dsRNA in a single step protocol.
• high throughput purification and analysis of a wide range of dsRNAs.
• developed IP RP HPLC for the rapid, high resolution analysis of the dsRNA.
• developed a novel method utilising RNase T1 for RNase mass mapping of dsRNA.

## 1 Introduction

RNA interference (RNAi) is a post-transcriptional inhibition of gene expression by a homology-dependent mRNA degradation mechanism evolved in many eukaryotes as a defence barrier against exogenous genetic materials such as transposons and viral genomes [1], [2]. RNAi utilises short, double-stranded (ds) RNAs termed small interfering RNAs (siRNA) to downregulate the expression of genes that contain complementary sequences. This mechanism was first discovered in *Caenorhabditis elegans* and subsequently in plants [3], [4].

The RNAi pathway is initiated by an enzyme called Dicer (a member of the RNase III family), which binds and cleaves long dsRNA molecules into short dsRNA fragments (siRNAs) of approximately 20 bp length. One strand of the siRNA duplex binds to Argonaute (Ago), an RNase H–like protein which represses translation of an mRNA on the basis of sequence complementarity [5].

In eukaryotes, endogenous siRNA pathways have many roles, including repressing repetitive and transposable genomic elements and defending the host against infection by RNA viruses. The RNAi pathway in insects includes several branches that function to silence the expression of both endogenous genes of the host and those of parasite and pathogen invaders. The exploitation of this pathway to block the expression of specific gene targets holds considerable promise for the development of novel RNAi-based insect management strategies [6]. In addition there are a wide number of future potential applications of RNAi to control agricultural insect pests as well as its use for prevention of diseases in beneficial insects [7].

The induction of RNAi by exogenous supply of dsRNA has been successful in a number of different organisms [3], [8]. Oral administration of dsRNA in pest insects has been demonstrated to induce RNAi and has significant potential utility for crop protection approaches [9], [10]. The size of the dsRNA product is also an important issue as short dsRNA have been reported to be limited in penetrance and expressivity [11]. Initial studies using *E. coli* BL21 (DE3) to generate dsRNA resulted in products that were degraded by RNase III to short, partially digested dsRNA [12], [13]. These short dsRNA molecules of about 12–15 bp in length are incapable of triggering RNA interference response in mammalian cells [14]. An RNase III-deficient *E. coli* strain HT115 (DE3) was engineered to produce intact specific dsRNA and when fed to *C. elegans* triggered strong interference [12].

Synthetic siRNA oligonucleotide duplexes or hairpin RNAs for RNAi studies have also been widely employed. However, a number of caveats are associated with these approaches including the costs associated with the synthesis of the oligonucleotide (often requiring specific modifications) and the time-consuming procedures including transfection and drug selection that are required for RNAi. The use of *in-vitro transcription or* bacteria to generate siRNAs can potentially reduce the cost of siRNAs and can provide a means of delivering siRNAs into cells. Large quantities of long dsRNAs can be produced by *in-vitro* transcription or in *E. coli* cells that lack RNase III with inducible T7 polymerase or φ6 RNA dependent RNA polymerase overexpression systems [11], [15], [16]. More recently, alternative approaches using a plant viral siRNA binding protein have been used to generate and purify siRNAs generated in bacteria [17]. However, for laboratory purposes we found it more practical to synthesise dsRNA using an inducible T7 polymerase overexpression system.

A variety of methods have been developed and utilised for the selective purification of dsRNA, including strategies utilising recombinant dsRNA-binding protein [18], phenol extraction in conjunction with cellulose affinity chromatography [19], [20] and the use of differential solubility of nucleic acids in LiCl to extract total dsRNA from viral infested tissues or cells [21]. A method based on anion exchange chromatography using convective interaction media (CIM) monolithic columns has also be used to separate dsRNA from ssRNA [22]. The resulting purified dsRNA is not readily amenable to conventional or next-generational sequencing techniques (NGS) or directly compatible with downstream mass spectrometry analysis. NGS approaches generate only short reads therefore does not readily provide important quantitative characterisation of large dsRNA.

The development of the analytical platform in this study enables the efficient and effective purification of dsRNA from bacterial cells prior to downstream RNAi applications. Furthermore, this approach enables relative quantification and characterisation of the dsRNA product providing high throughput analysis of the dsRNA, validation of the duplex nature of the dsRNA and characterisation of the dsRNA using mass spectrometry in conjunction with RNase mass mapping.

## 2 Materials and methods

### 2.1 Chemicals and reagents

Q5^®^ High-Fidelity DNA Polymerase, dNTPs, NTPS, designed primers from MWG Eurofins were used for PCR and in vitro transcription performed using HiScribe™ T7 High Yield RNA Synthesis Kit (New England Biolabs) and Synthetic gene from GeneArt^®^ Gene Synthesis (Invitrogen Life technologies). The *E. coli* strain, HT115(DE3) was obtained from Cold Spring Harbor Laboratory, NY, USA. Ampicillin sodium salt (Sigma-Aldrich), Tetracycline hydrochloride (Sigma-Aldrich), Isopropyl β-d-1-thiogalactopyranoside (IPTG) ≥99% (Sigma-Aldrich). TRIzol^®^ Max™ Bacterial RNA Isolation Kit with TRIzol^®^, Max Bacterial Enhancement Reagent (Life technologies) and the Ribopure™ bacterial RNA extraction kit (Life technologies) were used for RNA extractions. The WAVE System Transgenomic HPLC, Proswift RP-1S Monolith column (4.6 × 50 mm I.D. ThermoFisher) and buffers prepared with (TEAA) pH 7.0 (Fluka, UK), acetonitrile (ThermoFisher), HPLC grade water were used for nucleic acid analyses. RNase T1 (Ambion), RNase A (Ambion) and DNase I (Ambion) were used for purification and mass mapping of dsRNA. The Accucore C18 column (150 mm × 2.1 mm ID), the U3000 HPLC system (Thermo Scientific), and maXis Ultra High Resolution Time of Flight Instrument (Bruker Daltonics) were used in oligonucleotide analyses.

### 2.2 *In vitro* transcription of dsRNA

DNA was amplified from the plasmid pCOIV that contains a 765 bp sequence flanked on both sides with T7 promoter sequences and optimised synthetic T7 terminator sequences. PCR was performed using primers flanking the dsRNA gene using the following conditions. 0.02 U/μl Q5^®^ High-Fidelity DNA Polymerase, 200 μM dNTPs, 0.5 μM each of forward and reverse primer and 10 ng DNA template. The following PCR parameters were used: the initial denaturation was 1 cycle of 30 s at 98 °C, 30 cycles of 30 s at 98 °C, 30 s at 68 °C, and 30 s at 72 °C and a final extension at 72 °C for 2 mins. dsRNA and ssRNA were also generated using *in vitro* transcription in conjunction with HiScribe™ T7 High Yield RNA Synthesis Kit (New England Biolabs): 10 mM NTPs, 1 x reaction buffer, 1 μg DNA template and 2 μL HiScribe™ T7 polymerase in 20 μL RNase-free water.

### 2.3 Expression of dsRNA gene using *E. coli* HT115(DE3)

The *E. coli* strain, HT115(DE3) [12] was obtained from Cold Spring Harbor Laboratory, NY, USA. *E. coli* HT115 (DE3) cells were transformed with pCOIV. A colony from the transformed cells was inoculated into 5 mL LB media containing 10 ng/mL tetracycline and 100 μg/mL ampicillin and incubated overnight at 37 °C. This was followed by seeding 2 mL of the overnight culture into a 50 mL LB media containing the same concentration of antibiotics, incubated at 37 °C and allowed to reach an OD_600_ nm of 1. Then IPTG was added to the culture to 1 mM final concentration followed by further incubation at 37 °C for 3 h.

### 2.4 Extraction and purification of RNA

The total RNA was extracted from the cells using TRIzol^®^ Max™ Bacterial RNA Isolation Kit with TRIzol^®^ and Max Bacterial Enhancement Reagent (Life technologies) was used to extract total RNA following the manufacturer’s instructions. Briefly, the cells were harvested by centrifugation at 4500 rpm at 4 °C for 10 min. Approximately 10^8^ *E. coli* cells were suspended in pre-heated 200 μL Max Bacterial Enhancement Reagent and heated at 95 °C for 4 mins. 1 mL of TRIzol reagent was added, cells thoroughly mixed and incubated at room temperature for 5 min. Phenol-chloroform phase separation was achieved by adding 200 μL chloroform and incubating for 2 mins followed by centrifugation at 13,000 rpm for 5 min. The aqueous phase was transferred to a fresh 2 mL Eppendorf tube. The total RNA was precipitated out of solution by adding 1 mL isopropanol, incubating for 5 min and centrifuging at 14000 rpm for 7 min. The total RNA pellet was washed with 500 μL 70% ethanol and then dissolved in 100 μL distilled water. For purification of dsRNA, 4 U of DNase I (Ambion) was added and incubated for 10 min and the solution was adjusted to 300 mM NaCl and 3000 U of RNase T1 (Ambion) was added and incubated at 37 °C for 15 min. Then 560 μL PB buffer (Qiagen), 40 μL 5 M NaCl and 40 μL Isopropanol was added prior to solid phase extraction using silica-membrane column (Qiagen). After loading, the column was centrifuged at 13000 rpm for 1 min. The flow through was discarded and 700 μL PE (Qiagen) buffer added followed by centrifugation at 13,000 rpm for 1 min. The flow through was discarded and the centrifugation step repeated. The dsRNA was eluted with 100 μL distilled water. It is important to thoroughly desalt the dsRNA prior to digestions using RNase T1 in the presence of DMSO and heating the dsRNA. In addition, total RNA was extracted from cells using the Ribopure™ bacterial RNA extraction kit (Life technologies) following the manufacturer’s instructions.

### 2.5 Ion pair-reverse phase high performance liquid chromatography (IP-RP HPLC)

All samples were analysed by IP-RP-HPLC on a Transgenomic HPLC using a Proswift RP-1S Monolith column (4.6 × 50 mm I.D. ThermoFisher). Chromatograms were analysed using UV detection at a wavelength of 260 nm. The chromatographic analysis was performed using the following conditions: Buffer A 0.1 M triethylammonium acetate (TEAA) pH 7.0 (Fluka, UK); Buffer B 0.1 M TEAA, pH 7.0 containing 25% acetonitrile (ThermoFisher). RNA was analysed using the following gradients. Gradient (1) starting at 20% buffer B the linear gradient was extended to 22% buffer B in 2 min, followed by a linear extension to 52% buffer B over 15 min, followed by a linear extension to 65% buffer B over 2.5 min at a flow rate of 1.0 mL/min at 50 °C and 75 °C. Gradient (2) starting at 35% buffer B the linear gradient was extended to 55% buffer B in 3 min, followed by a linear extension to 65% buffer B over 10 min, isocratic at 65% buffer B for 2.5 mins and a linear extension to 100% buffer B over 2.5 min at a flow rate of 1.0 mL/min at both non denaturing (50 °C) and denaturing conditions (75 °C).

### 2.6 Agarose gel electrophoresis

1% agarose gel was used for gel electrophoresis. RNA loading dye 2x (NEB) was added to RNA sample and loaded on the gel. 1X TAE buffer (40 mM Tris (pH 7.6), 20 mM acetic acid and 1 mM EDTA) was used to perform electrophoresis at 100 V for 45 min. The agarose gel was pre-stained with ethidium bromide and UV imaging system fitted with charge coupled device (CCD) camera (Biospectrum^®^ Multispectral Imaging System).

### 2.7 Digestion of ssRNA using RNase A/T1 and DNase I

RNase T1 digests was performed using a ratio of 1000 U of RNase T1 to 1 μg RNA in RNase-free water or RNase free water + 0.5 M NaCl for 15 mins at 37 °C. The RNase A assay was performed using a ratio 10 ng RNase A per 1 μg RNA in RNase-free water for 15 min at 37 °C. Removal of DNA was performed using 2 U DNase I to 1 μg RNA.

### 2.8 RNase mass mapping

Following purification, 5 μg of dsRNA in RNase-free water was incubated with 100 ng RNase A at 37 °C for 30 min. For RNase T1 mass mapping, 5 μg of purified dsRNA in 50% DMSO was incubated at 90 °C for 30 s and allowed to cool to room temp. 2000 U RNase T1 was added and reaction mix incubated for 15 min at 37 °C.

Subsequently, 2.5 μg, was analysed using LC ESI MS using an Accucore C18 column (150 mm × 2.1 mm ID). LC MS buffer A: 80 mM 1,1,1,3,3,3,-Hexafluoro-2-propanol (HFIP, Sigma-Aldrich) with 20 mM TEAA (Sigma-Aldrich), LC MS buffer B: LC MS buffer A with 50% acetonitrile (v/v) (ThermoFisher). Starting with 10% buffer B, an isocratic step was performed for 2 mins followed by a linear extension to 20% B in 20 mins, then 20%B to 30% B over 10 min and 30%B to 90%B for 3 mins at a flow rate of 100 μL min^−1^. Mass spectrometry analysis was performed using a maXis Ultra High Resolution Time of Flight Instrument (Bruker Daltonics).

Data acquisition was performed in negative ion mode with a selected mass range of 300−2500 *m*/*z*. Ionisation voltage of − 2000 V was used to maintain capillary current between 30 and 50 nA. Temperature of nitrogen 300 °C at a flow rate of 6.0 L/h and N_2_ nebuliser gas pressure at 0.4 bar. A list of theoretical monoisotopic masses of oligoribonucleotides was generated using Mongo Oligo Mass Calculator (http://mods.rna.albany.edu/masspec/Mongo-Oligo). The output of this analysis produces a theoretical sequence ladder of oligoribonucleotides for the dsRNA with all possible chemical termini including; 5′-OH, −phosphate, −cyclic phosphate and 3′-OH, −phosphate, −cyclic phosphate. Mongo Oligo Mass Calculator was also used to predict the CID fragment ions of the theoretical oligoribonucleotides.

## 3 Results and discussion

### 3.1 Extraction of dsRNA from *E. coli*

Common approaches used to extract RNA from bacterial cells are centred on methods that combine guanidinium thiocyanate and phenol/chloroform extractions [23]. A variety of methods have been employed for the extraction of RNA from bacterial cells, however these methods can often result in low yields or low quality total RNA [24]. Improvements in the quality and quantity of RNA extracted from bacterial cells were achieved using hot SDS/hot phenol isolation techniques. However these approaches required DNase treatment prior to downstream gene expression analysis [25]. More recently, alternative approaches using formamide-based RNA extractions from bacterial cells were developed to efficiently extract total RNA from bacterial cells [26]. This method termed (RNAsnap™) generated similar quality and yield compared to the commercial guanidium isothiocyanate −phenol/chloroform based methods. In addition phenol/chloroform free methods have also been developed for the efficient extraction of RNA [27]. Therefore, optimising efficient extraction of high quality RNA whilst simultaneously minimising RNA degradation and rapidly assessing RNA quality is important.

**Fig. 1:**
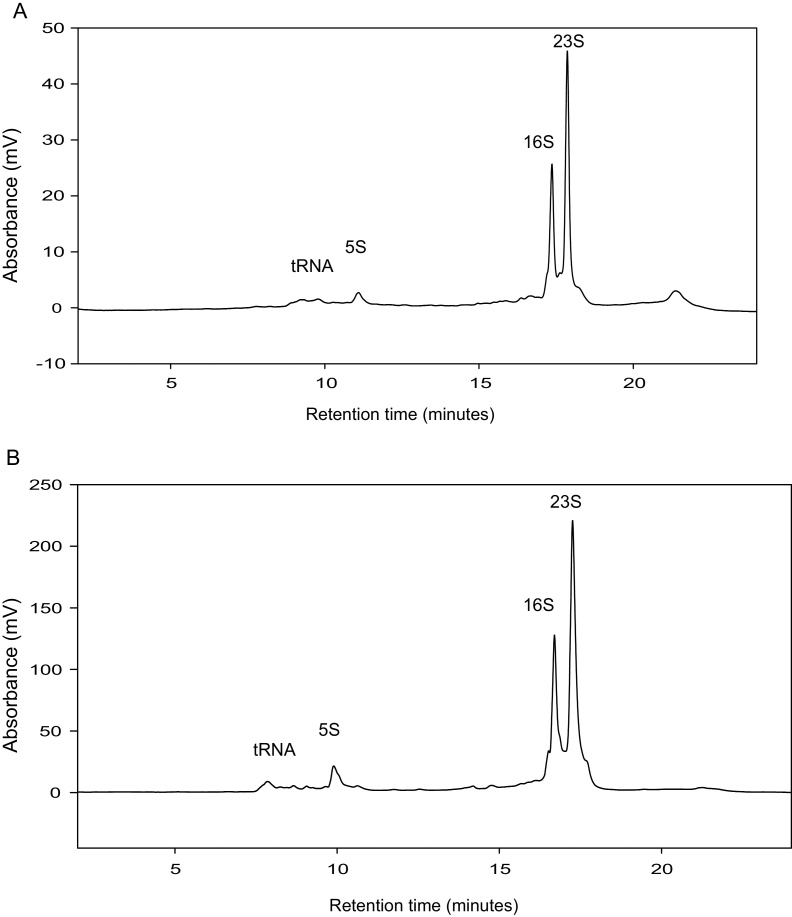
IP RP HPLC chromatograms of total RNA extracted from *E. coli* using Ribopure™ bacterial RNA extraction kit. (a) IP RP HPLC chromatogram of total RNA from E. coli HT115 (DE3) cells transformed with plasmid pCOIV prior to induction. (b) IP RP HPLC chromatogram of total RNA from *E. coli* HT115 (DE3) cells transformed with plasmid pCOIV following induction with IPTG. The corresponding 5S, 16S and 23S rRNA are highlighted. 2 and 8 μg of total RNA was analysed using gradient condition 1 at 260 nm UV detection.

**Fig. 2:**
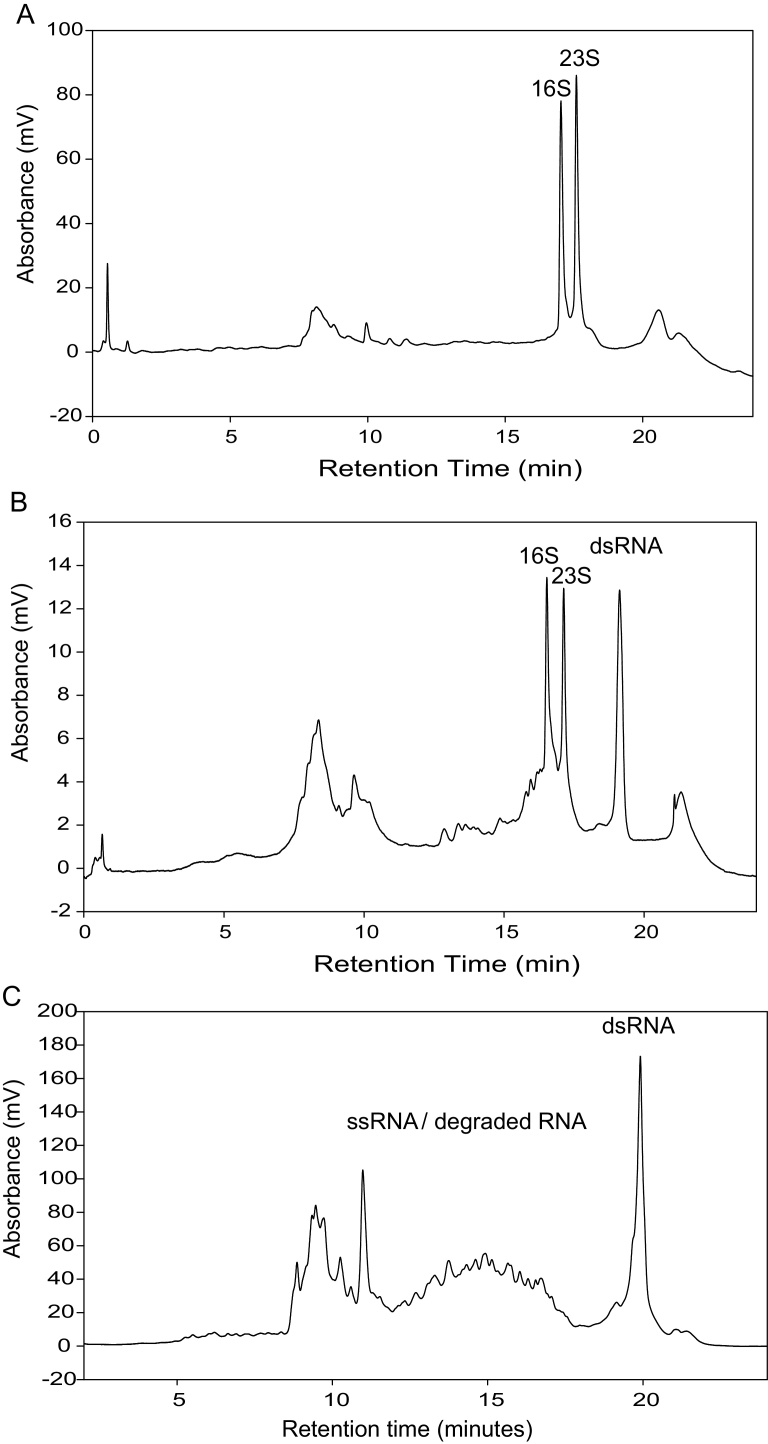
IP RP HPLC chromatograms of total RNA extracted from *E. coli* using TRIzol^®^ Max™ Bacterial RNA Isolation Kit. (a) IP RP HPLC chromatogram of total RNA from *E. coli* HT115 (DE3) cells transformed with plasmid pCOIV prior to induction. (b) IP RP HPLC chromatogram of total RNA from *E. coli* HT115 (DE3) cells transformed with plasmid pCOIV following induction with IPTG. The corresponding rRNA and dsRNA are highlighted. Approximately 15 μg of total RNA was analysed using gradient condition 1 at 260 nm UV detection. (c) IP RP HPLC chromatogram of total RNA extracted from *E. coli* HT115 (DE3) in conjunction with mechanical shearing and TRIzol. Approximately 10 μg of total RNA was analysed using gradient condition 1 at 260 nm UV detection.

*E. coli* HT115 (DE3) cells were transformed with plasmid pCOIV to express a dsRNA (∼756 bps). Following induction and transcription of the dsRNA in *E. coli* we evaluated a number of alternative commercially available extraction methods including TRIzol^®^ Max™ Bacterial RNA Isolation Kit (TRIzol*^®^* reagent) and the Ribopure™ bacterial RNA extraction kit (RNAwiz reagent) to extract the dsRNA. Following extraction of the dsRNA, analysis was performed using ion pair reverse phase liquid chromatography (IP-RP HPLC). The results showed the RiboPure™ RNA Purification Kit-bacteria kit efficiently extracted high quality ribosomal RNA/ssRNAs but no dsRNA was observed (see Fig. 1a/b). RNA extracted from the *E. coli* cells using TRIzol^®^ (TRIzol^®^ Max™ Bacterial RNA Isolation Kit) and analysed using IP RP HPLC is shown in Fig. 2. The results also show that the rRNAs (rRNAs) are efficiently extracted with minimal degradation. In contrast to using RNAwiz, high yields of dsRNA were also obtained.

For the extraction of dsRNA, optimising the extraction methods to ensure minimal degradation of the ssRNA (rRNA) is not necessarily a stringent requirement in contrast to downstream gene expression analysis using qRT PCR or the preparation of RNAseq libraries. The additional stability conferred by the dsRNA over ssRNA is advantageous during extraction. An example is shown in Fig. 2c where using mechanical shearing in conjunction with TRIzol reagent to extract the dsRNA resulted in rRNA degradation during the procedure. However, the results show that extraction of the dsRNA has not been significantly affected.

### 3.2 Purification of dsRNA

**Fig. 3:**
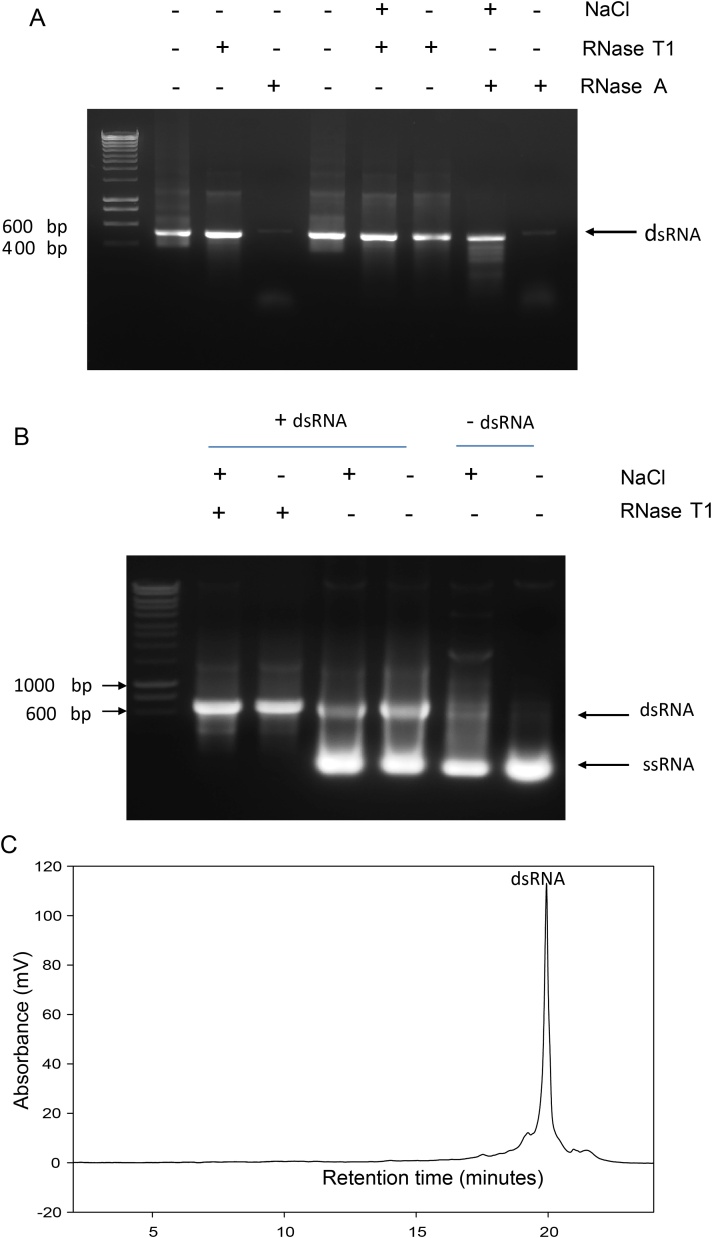
Purification of dsRNA from *E. coli*. (a) Agarose gel electrophoresis of purified *in vitro* transcribed dsRNA and ssRNA. Following *in vitro* transcription, 2 μg of dsRNA containing excess ssRNA were incubated with RNase T1 in both the presence and absence of 0.5 M NaCl and purified by SPE. (b) Agarose gel electrophoresis of extracted and purified dsRNA. Following TRIzol extraction of total RNA from *E. coli* expressing dsRNA samples were incubated with RNase T1 in both the presence and absence of 0.3 M NaCl as indicated prior to purification using solid phase extraction. Control experiments were performed using total RNA from non-induced *E. coli*. (c) IP RP HPLC chromatogram of purified dsRNA. Following TRIzol extraction the total RNA from *E. coli* was purified in a single step using RNase T1/DNase in conjunction with solid phase extraction prior to analysis using IP RP HPLC gradient condition 1 at 260 nm UV detection.

Following extraction of total RNA from *E. coli* cells expressing the dsRNA using the TRIzol*^®^* reagent, further purification methods were optimised in an effort to remove contaminating RNA (tRNA/rRNA) and DNA. Current strategies to isolate dsRNA use phenol-chloroform extractions in conjunction with RNase A/DNase to remove contaminating ssRNA and DNA [12]. We have observed that at very low salt concentrations dsRNA is more resistant to RNase T1 than to RNase A, (see Fig. 3a). The result suggests that dsRNA may be relatively susceptible to RNase A digestion under low salt concentrations, suggesting that RNase T1 is not only better suited for the selective degradation of ssRNA species than RNase A but also for optimal dsRNA quality and yield.

Following extraction of total RNA from *E. coli* cells expressing dsRNA using TRIzol*^®^* reagent extraction in conjunction with gel electrophoresis analysis, we demonstrated that RNase T1 selectively degrades ssRNA (rRNAs) without degrading the dsRNA in both the presence or absence of 0.3 M NaCl (see Fig. 3b) which is consistent with previous observations that dsRNA is unaffected by RNase T1 [28]. Control experiments are shown using total RNA from non-induced *E. coli* cells.

Therefore, in an approach to purify the dsRNA from total RNA extracted from *E. coli* and minimise the degradation of dsRNA, an optimised method was developed using RNase T1/DNase I treatment in conjunction with solid phase extraction using a silica membrane spin column to further purify the dsRNA from contaminating degraded ssRNA/DNA, salts and phenol/choloroform. Fig. 3c shows the IP-RP HPLC analysis of the purified dsRNA from *E. coli* cells expressing the dsRNA. The results demonstrate the successful purification of dsRNA from the *E. coli* cells expressing dsRNA from contaminating ssRNA (rRNA/tRNA) and their associated degradation products.

### 3.3 Ion pair reverse phase HPLC analysis of dsRNA

In order to validate the successful purification of the dsRNA from *E. coli* prior to downstream RNAi studies, it is important to analyse the dsRNA. A number of important parameters including the homogeneity, presence of impurities, validation of dsRNA (vs ssRNA) and sequence of the dsRNA product should be measured as part of the analytical platform. Ion pair reverse phase high-performance liquid chromatography (IP RP HPLC) on nonporous alkylated poly(styrene-divinylbenzene) particles combines very high-resolution separation of nucleic acids under both non-denaturing and denaturing conditions with very short analysis times, therefore offering significant advantages and opportunities for the analysis of RNA compared to gel electrophoresis [29]. In addition, the advantages of chromatographic methods for the purification of siRNA and dsRNA has also been demonstrated using anion exchange chromatography using convective interaction media monolithic columns [30].

**Fig. 4:**
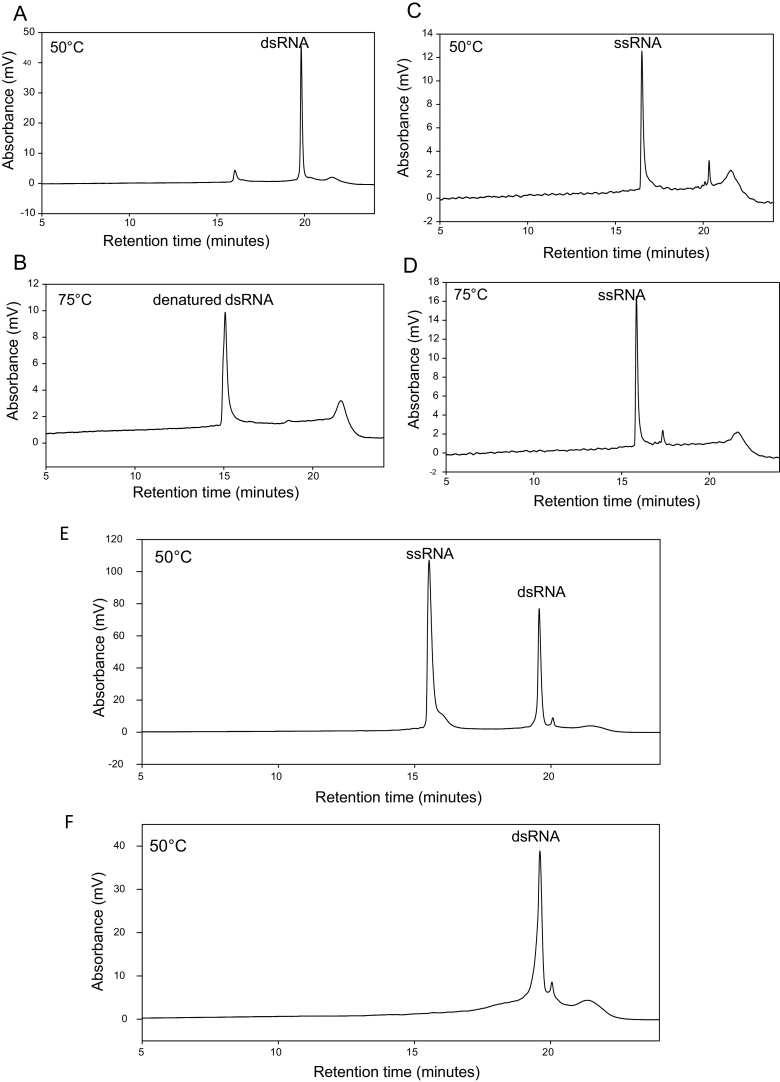
IP RP HPLC analyses of dsRNA and ssRNA under denaturing and non-denaturing conditions. (a) IP RP HPLC chromatogram of dsRNA at 50 °C. (b) IP RP HPLC chromatogram of dsRNA at 75 °C. (c) IP RP HPLC chromatogram of ssRNA at 50 °C. (d) IP RP HPLC chromatogram of ssRNA at 75 °C. (e) IP RP HPLC chromatogram of *in vitro* transcribed dsRNA with an excess of ssRNA. (f) IP RP HPLC chromatogram of purified dsRNA. Following *in vitro* transcription, the RNA was purified in a single step using RNase T1/DNase in conjunction with solid phase extraction. 2–3 μg of in vitro transcribed ds/ssRNA was analysed using gradient 1 at 260 nm UV detection.

To validate the presence of dsRNA, the RNA expressed in *E. coli* was analysed using IP RP HPLC under non-denaturing conditions at 50 °C. The analysis of dsRNA and ssRNA generated from the same template DNA using *in vitro* transcription is shown in Fig. 4a–d. The results show that only a small change in the retention time is observed for the ssRNAs analysed at 50 °C and 75 °C. In contrast, a large change in the retention time is observed for duplex RNA when comparing the IP RP HPLC analysis at 75 °C and 50 °C. Analysis of the dsRNA at 75 °C effectively denatures the duplex dsRNA into the corresponding two ssRNAs, which elute at the expected earlier retention time compared to the dsRNA. Therefore, analysis of the duplex dsRNA at 50 °C provides a rapid quantitative measurement of the relative amount of duplex dsRNA generated. Further analysis at 75 °C demonstrates the dissociation of the dsRNA strands, providing additional validation of the duplex nature of the dsRNA. Further examination in this case reveals only a small amount of ssRNA is present from the *in vitro* transcription (see Fig. 4a–d). The relative differences in the hydrophobicity of the duplex and ssRNA species results in the different retention times using IP RP HPLC consistent with previous observations ([25], Noll et al., 2011).

It is interesting to note that often during *in vitro* transcription reactions using high yield T7 polymerases IP RP HPLC analysis revealed an excess of ssRNA compared to dsRNA (see Fig. 4e). Relative quantification of the dsRNA was calculated from the IP RP HPLC by first determining the relative peak areas of the same dsRNA (500 ng) analysed at both 50 °C and 75 °C. By measuring the peak areas of the dsRNA and ssRNAs, this enables us to accurately account for the hypochromicity of the dsRNA (see Supplementary Fig. S1a). Validation of the accuracy of the IP RP HPLC quantification was subsequently performed by mixing known amounts of the ssRNA and dsRNA standards generated using *in vitro* transcription. The standards were analysed using IP RP HPLC, peak areas measured and the correction factor determined above was applied to determine the relative% of dsRNA. The results show good agreement with calculated ratios of 51.9% and 68.9% compared with expected ratios of 50% and 66.67% respectively (see Supplementary Fig. S1 b/c). From the chromatogram shown in Fig. 4e using the method described, it was determined that 39.6% of the RNA generated from *in vitro* transcription was dsRNA. These results highlight the importance of analysing the relative proportion of ssRNA and dsRNA using this approach prior to downstream applications of the dsRNA. The *in vitro* transcribed material in which an excess of ssRNA was present was further subjected to RNase T1 prior to analysis using IP RP HPLC (See Fig. 4f). The results further verify the ability to completely remove the contaminating ssRNA in conjunction with IP RP HPLC analysis to rapidly separate the duplex dsRNA from its corresponding ssRNAs. The results demonstrate that IP RP HPLC can rapidly analyse the dsRNA product, determine the purity of the product and importantly gives information on the proportions of ssRNA and dsRNA present. Furthermore, the application of IP RP HPLC could also be extended to further purify the dsRNA if required from contaminating salts, NTPs, ssRNA/dsRNA, and proteins.

To further validate the duplex nature of the purified dsRNA, the temperature-dependent dissociation between the two RNA-strands was measured using both IP RP HPLC and the binding of the duplex-intercalating fluorophore SYBR green (see supplementary Fig. S2-4). The results confirm the duplex nature of the purified dsRNA where typical dissociation characteristics were observed using both IP RP HPLC and SYBR green fluorescence intensity measurements. In contrast, as expected no temperature-dependent dissociation was observed for ssRNA (see supplementary Fig. S2-4).

### 3.4 Characterisation of dsRNA using RNase mass mapping

Mass spectrometry has emerged as an increasingly powerful tool for the identification and characterisation of nucleic acids. Prior to mass spectrometry analysis, the RNA of interest is routinely first purified using HPLC [31], [32], [33], [34], [35]. This often large biomolecule is subsequently digested with endonucleases into smaller oligoribonucleotides that are more amenable for chromatographic separation and intact mass measurements [36], [37]. Additional sequence information from the oligoribonucleotides can be obtained using tandem mass spectrometry (MS/MS) *via* collision induced dissociation (CID) or post source decay (PSD) [38]. This approach has been successfully employed to characterise a wide range of RNAs [31], [37], [39], [40], [41], [42]. Ribonuclease (RNase) mapping of dsRNA is limited by the availability of known dsRNA base specific RNases. Whilst RNase A has previously been shown to cleave dsRNA under specific ionic strength conditions, there is limited information regarding the specificity under these conditions. Moreover, RNase T1 is not active against dsRNA [28], [43].

**Fig. 5:**
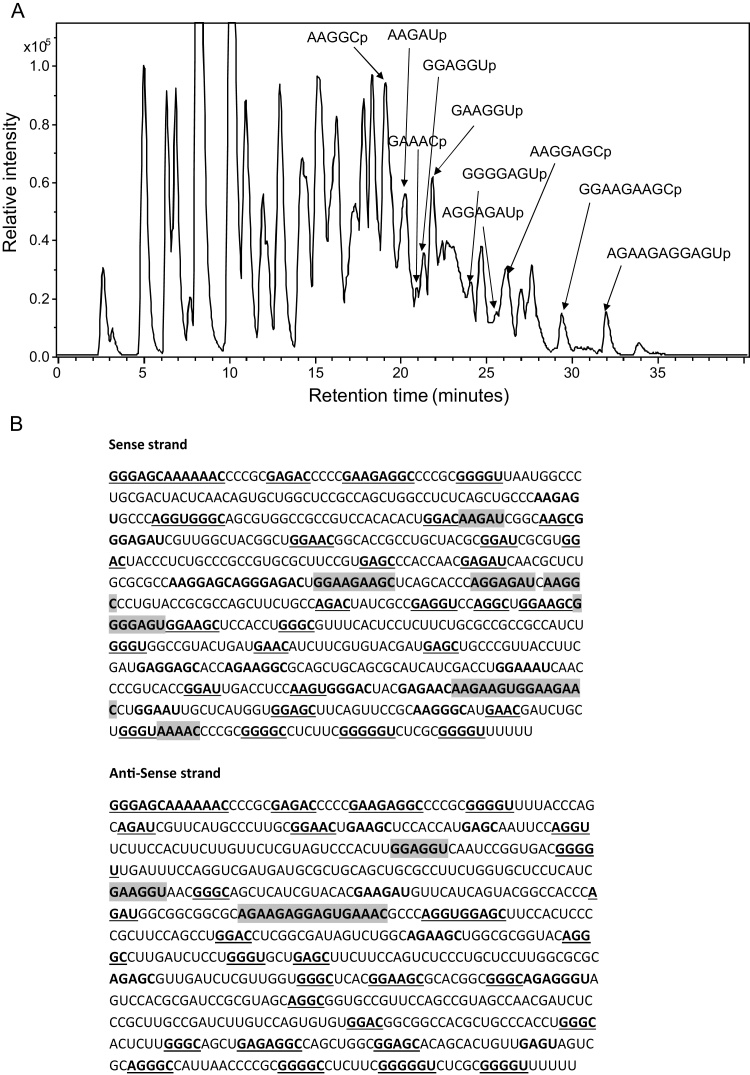
RNase A Mass Mapping of the dsRNA using LC ESI MS. (a) Base peak chromatogram of the oligoribonucleotides generated from an RNase A digest of the purified dsRNA. Analysis was performed using LC ESI MS on a maXis ultra-high resolution time of flight instrument. A number of the identified oligoribonucleotides are highlighted. (b) Summary of the RNase mass mapping. Underlined bold = monoisotopic masses correspond to a number of theoretical sequence isomers in either sense or antisense strand. Bold = monoisotopic masses corresponding to a number of theoretical sequence isomers that are unique in either sense or antisense strand. Grey highlight = monoisotopic masses corresponding to single predicted unique oligoribonucleotide sequence in only the sense or antisense strand.

Here we have optimised the analysis of dsRNA using RNase digestion prior to liquid chromatography interfaced with mass spectrometry analysis. To our knowledge, RNase mass mapping approaches have only previously been employed to analyse ssRNA. Expression of a 765 bp dsRNA in *E. coli* and purification was performed as outlined previously. Subsequently the purified dsRNA was digested with RNase A under optimised conditions and the resulting oligoribonucletides were analysed using LC ESI MS in an approach to further characterise the dsRNA. Following LC ESI MS analysis (see Fig. 5a) the identified oligoribonucleotides resulting from the monoisotopic masses obtained were compared to the theoretical monoisotopic masses expected from an *in silico* RNase A digest of the dsRNA. Analysis of the RNase mass mapping data shows for the first time that RNase A digestion specifically digests the dsRNA 3′ of U and C residues (similar to its specificity in ssRNA). A map of all the identified oligoribonucleotides for both the sense and antisense strands of the dsRNA is summarised in Fig. 5b and supplementary Fig. S5. For clarity the identified short oligoribonucleotides ≤3 mers are not included. Due to the complexity of the sample, a number of oligoribonucleotide monoisotopic masses obtained could be assigned to a number of different oligoribonucleotide sequences generated from the RNase digest in either the sense or antisense strands. However a number of monoisotopic masses were identified that correspond to unique oligoribonucleotides including the oligoriobonucleotides AAGAUp (1656.263 Da) and GAAGGUp (2017.320 Da) in the sense and antisense strands respectively. The corresponding MS and tandem MS data is shown in supplementary Fig. S6/7 providing further verification of the unique oligoribonucleotides in the sense and antisense strands of the dsRNA. The RNase mass mapping identified all of the theoretical RNase A oligoribonucleotides (≥4 mers) generated from the 765 bp dsRNA. The RNase A mass mapping of the dsRNA in conjunction with the tandem MS analysis of a number of unique oligoriobonucleotides provides further evidence for the identification of the corresponding dsRNA sequence.

**Fig. 6:**
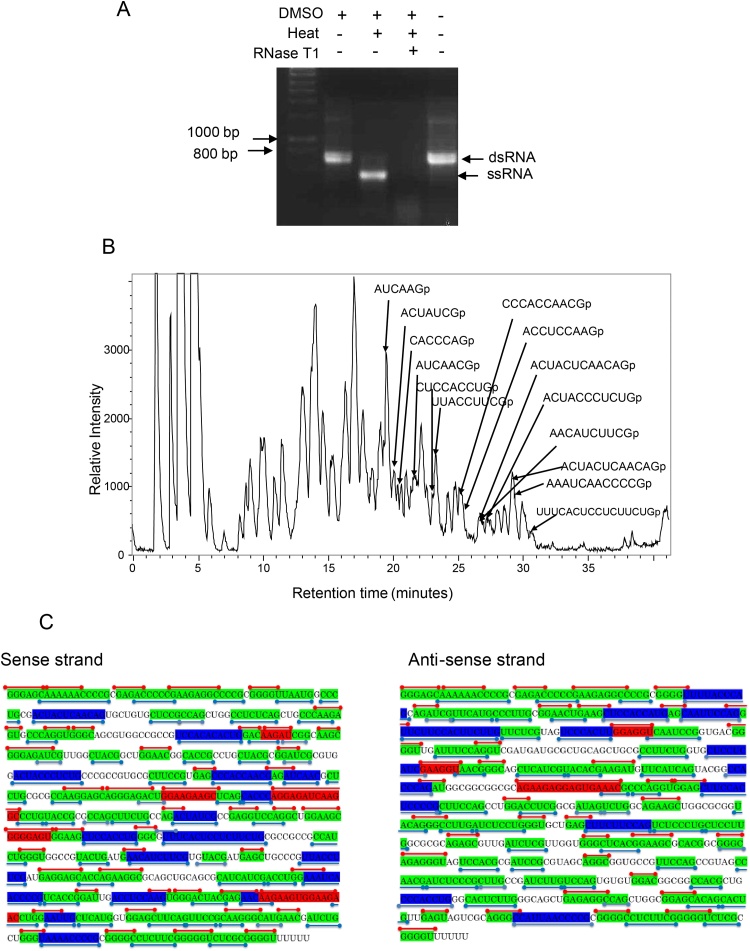
RNase T1 Mass Mapping of the dsRNA using LC ESI MS. (a) Base peak chromatogram of the oligoribonucleotides generated from an RNase T1 digest of the purified dsRNA. Analysis was performed using LC ESI MS on a maXis ultra-high resolution time of flight instrument. A number of the identified oligoribonucleotides are highlighted. (b) Summary of the combined RNase A/T1 mass mapping. RNase A fragments are shown in red lines and RNase T1 in blue. Sequences highlighted in green = monoisotopic masses corresponding to a number of theoretical sequence isomers in either sense or antisense strand. Blue = monoisotopic masses corresponding to single predicted unique oligoribonucleotide sequence in only the sense or antisense strand from the RNase T1 digest. Red = monoisotopic masses corresponding to single predicted unique oligoribonucleotide sequence in only the sense or antisense strand from the RNase A digest. (For interpretation of the references to colour in this figure legend, the reader is referred to the web version of this article.)

In an approach to improve the sequence coverage of dsRNA using RNase mass mapping, we developed a protocol for RNase T1 mass mapping of dsRNA. Although dsRNA is resistant to RNase T1 [28] it was proposed that following denaturation of the dsRNA into ssRNA under conditions that retained RNase T1 activity this approach would enable base specific cleavage of the dsRNA. A range of denaturing reagents including urea and guanadinium hydrochloride were used in conjunction with RNase T1 without success (data not shown). In contrast, the addition of RNase T1 following the heating of dsRNA in the presence of DMSO resulted in efficient cleavage of the dsRNA (see Fig. 6a). Control experiments in the absence of RNase T1 demonstrate that under these conditions the dsRNA is effectively denatured to ssRNA (see Fig. 6a). Following optimisation of the above method RNase T1 digest of the 765 bp dsRNA was performed using LC ESI MS analysis (see Fig. 6b). Following LC ESI MS analysis the identified oligoribonucletides resulting from the monoisotopic masses obtained were compared to the theoretical monoisotopic masses expected from an *in silico* RNase T1 digest of the dsRNA. Analysis of the RNase mass mapping data shows that RNase T1 digestion specifically digests the dsRNA 3′ side of G. A map of all the identified oligoribonucleotides for both the sense and antisense strands of the dsRNA is shown in supplementary Fig. S8. For clarity the identified short oligoribonucleotides ≤3 mers are not included. Following RNase mass mapping of the dsRNA using both RNase A/T1 the combined RNase mass map is shown in Fig. 6c. Using this combined approach over 82% and 77% sequence coverage was obtained based on the identification of oligoribonucleotides (>3mers) for the sense and antisense strands respectively.

## 4 Conclusions

We have developed a range of analytical tools that enable the high throughput purification and characterisation of dsRNA. In this study we have optimised standard, commercially available TRIzol extractions in conjunction with a single step protocol to remove contaminating DNA and ssRNA during the purification procedure. In addition, we have utilised and developed IP RP HPLC for the rapid, high resolution analysis of the dsRNA. This approach enables accurate sizing of the dsRNA, and further verification of the purity of the duplex dsRNA product over contaminating rRNAs and corresponding ssRNAs. These combined approaches enable the high throughput purification and analysis of a wide range of dsRNAs generated either via bacterial expression systems or *in vitro* transcription. In addition, we have developed and optimised RNase mass mapping approaches using RNase A and novel methods in conjunction with RNase T1 to further characterise dsRNA in conjunction with liquid chromatography interfaced with mass spectrometry analysis. The application of robust analytical methods to rapidly assess product quality following the purification of the dsRNA product from impurities including contaminating RNAs combined with methods to characterise and identify the dsRNA products are important requirements prior to the downstream application of dsRNA for RNAi studies. The development of RNase mass mapping approaches to characterise dsRNA is also hugely important to RNAi applications as dsRNA is not readily amenable to conventional or next-generational sequencing techniques (NGS). NGS approaches generate only short reads therefore does not readily provide important quantitative characterisation of large dsRNA.
