# Supplementary material for: Purification and characterisation of dsRNA using ion pair reverse phase chromatography and mass spectrometry
Source: J Chromatogr A. 2017 Feb 10;1484:14–25. doi: 10.1016/j.chroma.2016.12.062 (PMC5267946; doi:10.1016/j.chroma.2016.12.062)
Supplement: Supplementary file 1 [file mmc1.pptx]

## Slide 1
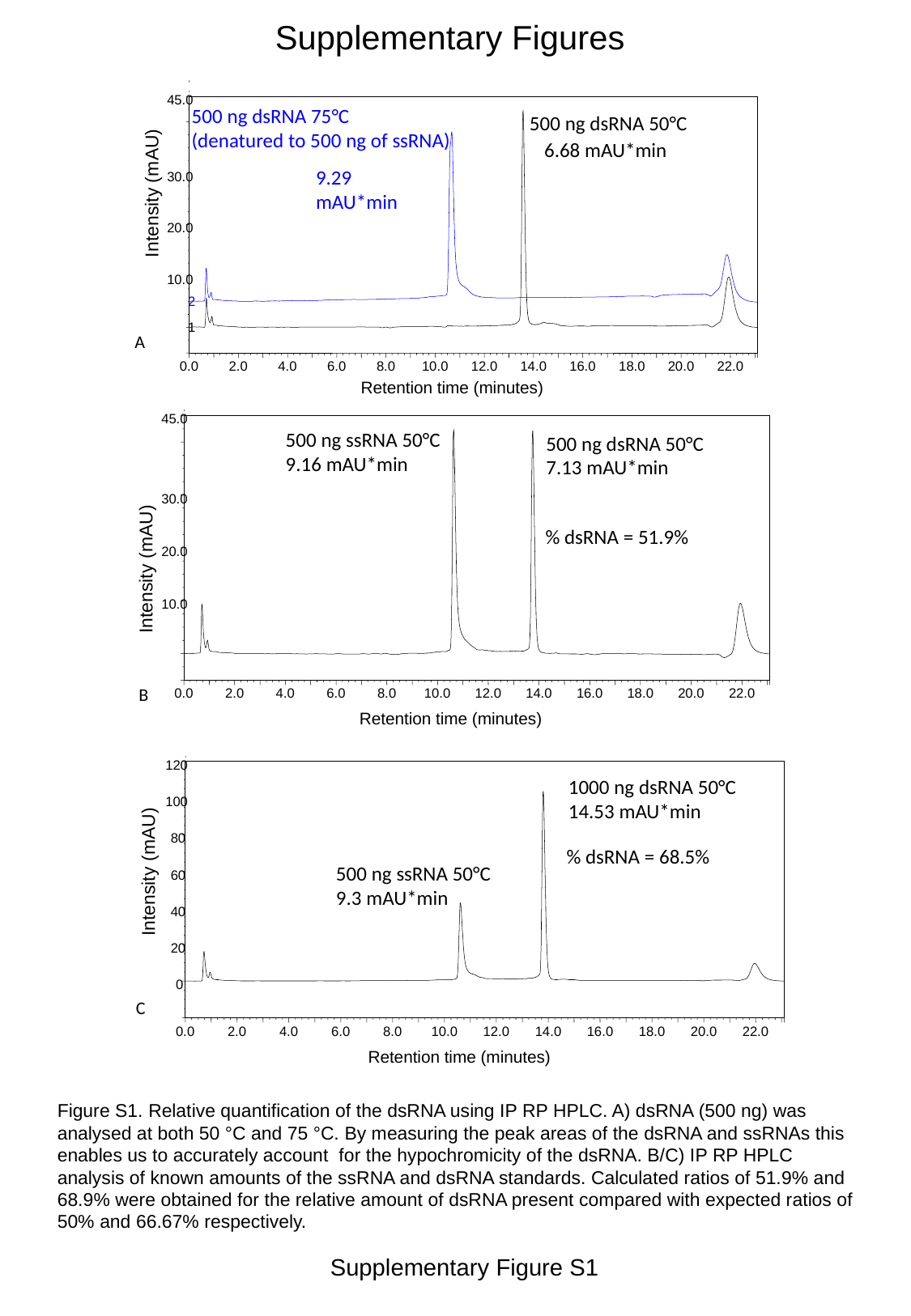

Supplementary Figures
45.0
6.68 mAU*min
9.29 mAU*min
30.0
20.0
10.0
2
1
0.0
2.0
4.0
6.0
8.0
10.0
12.0
14.0
16.0
18.0
20.0
22.0
Intensity (mAU)
Retention time (minutes)
500 ng dsRNA 75°C
(denatured to 500 ng of ssRNA)
500 ng dsRNA 50°C
A
45.0
30.0
20.0
Intensity (mAU)
10.0
0.0
2.0
4.0
6.0
8.0
10.0
12.0
14.0
16.0
18.0
20.0
22.0
500 ng ssRNA 50°C
9.16 mAU*min
500 ng dsRNA 50°C
7.13 mAU*min
% dsRNA = 51.9%
B
120
100
80
Intensity (mAU)
60
40
20
0
0.0
2.0
4.0
6.0
8.0
10.0
12.0
14.0
16.0
18.0
20.0
22.0
1000 ng dsRNA 50°C
14.53 mAU*min
% dsRNA = 68.5%
500 ng ssRNA 50°C
9.3 mAU*min
C
Retention time (minutes)
Retention time (minutes)
Figure S1. Relative quantification of the dsRNA using IP RP HPLC. A) dsRNA (500 ng) was analysed at both 50 °C and 75 °C. By measuring the peak areas of the dsRNA and ssRNAs this enables us to accurately account for the hypochromicity of the dsRNA. B/C) IP RP HPLC analysis of known amounts of the ssRNA and dsRNA standards. Calculated ratios of 51.9% and 68.9% were obtained for the relative amount of dsRNA present compared with expected ratios of 50% and 66.67% respectively.
Supplementary Figure S1

## Slide 2
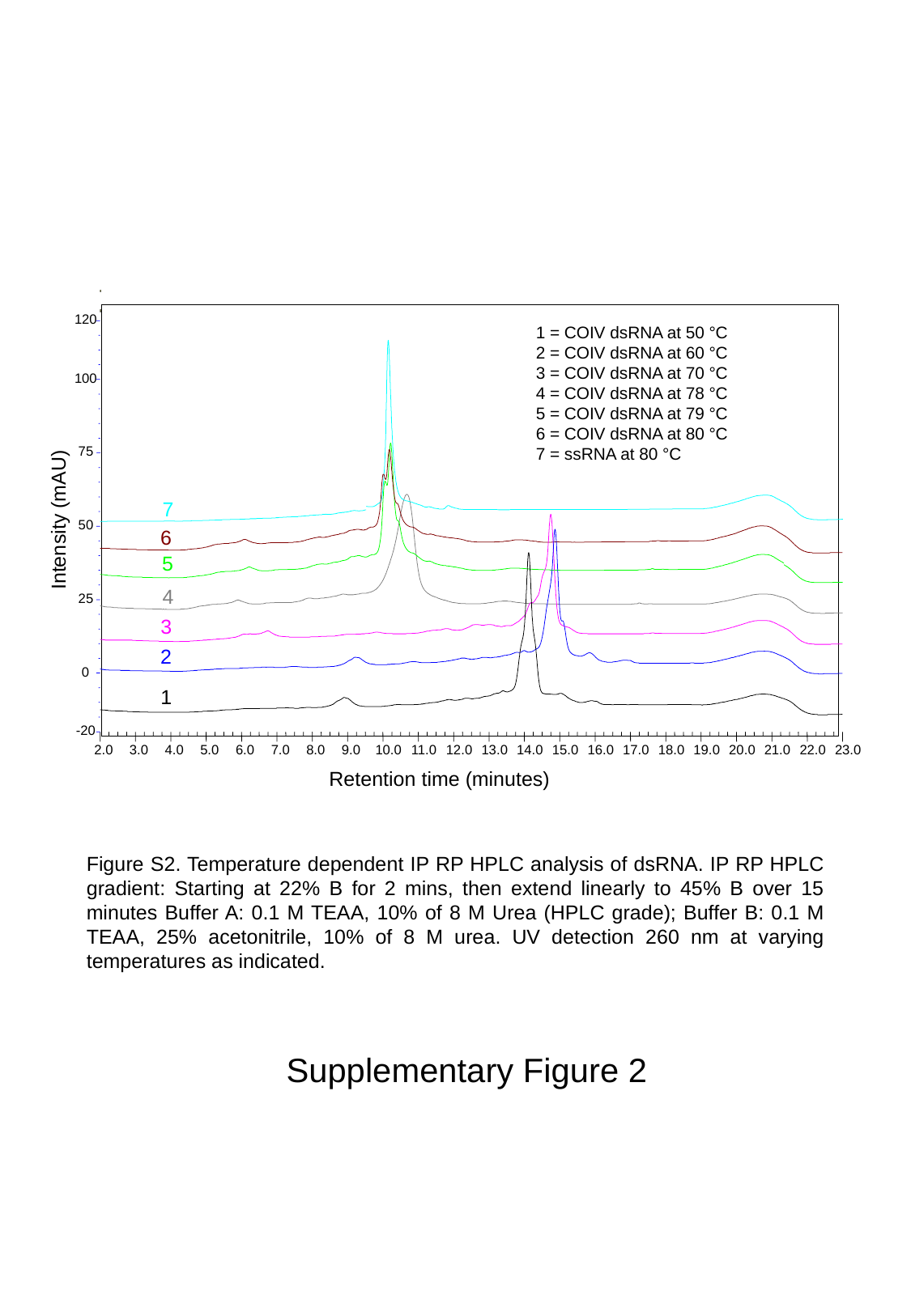

120
1 = COIV dsRNA at 50 °C
2 = COIV dsRNA at 60 °C
3 = COIV dsRNA at 70 °C
4 = COIV dsRNA at 78 °C
5 = COIV dsRNA at 79 °C
6 = COIV dsRNA at 80 °C
7 = ssRNA at 80 °C
100
75
Intensity (mAU)
7
50
6
5
4
25
3
2
0
1
-20
2.0
3.0
4.0
5.0
6.0
7.0
8.0
9.0
10.0
11.0
12.0
13.0
14.0
15.0
16.0
17.0
18.0
19.0
20.0
21.0
22.0
23.0
Retention time (minutes)
Figure S2. Temperature dependent IP RP HPLC analysis of dsRNA. IP RP HPLC gradient: Starting at 22% B for 2 mins, then extend linearly to 45% B over 15 minutes Buffer A: 0.1 M TEAA, 10% of 8 M Urea (HPLC grade); Buffer B: 0.1 M TEAA, 25% acetonitrile, 10% of 8 M urea. UV detection 260 nm at varying temperatures as indicated.
Supplementary Figure 2

## Slide 3
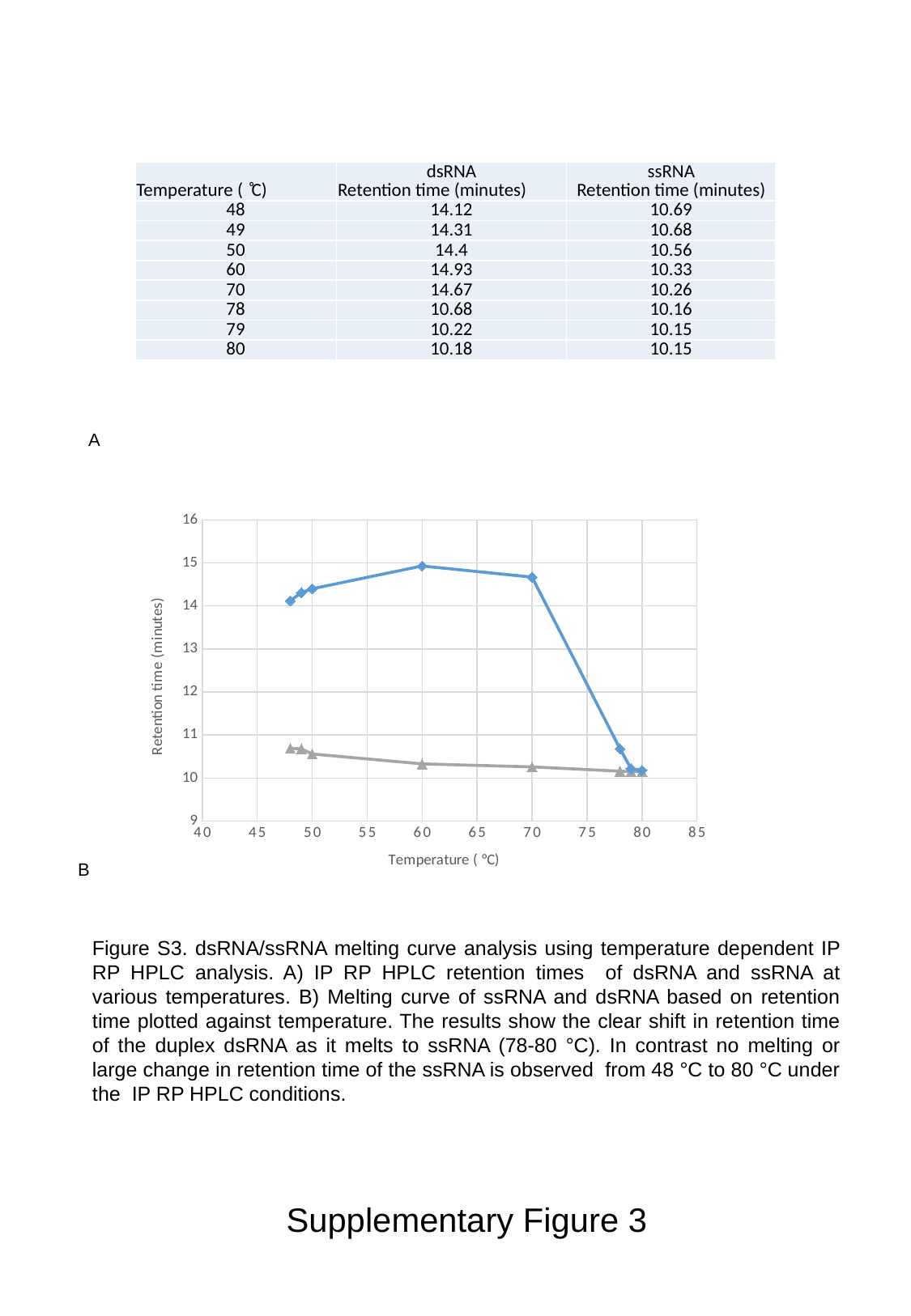

| Temperature ( ͦC) | dsRNA Retention time (minutes) | ssRNA Retention time (minutes) |
| --- | --- | --- |
| 48 | 14.12 | 10.69 |
| 49 | 14.31 | 10.68 |
| 50 | 14.4 | 10.56 |
| 60 | 14.93 | 10.33 |
| 70 | 14.67 | 10.26 |
| 78 | 10.68 | 10.16 |
| 79 | 10.22 | 10.15 |
| 80 | 10.18 | 10.15 |
A
### Chart
| Category | Retention time (min) dsRNA | Temperature ( ͦC) ssRNA | Retention time (min) ssRNA |
|---|---|---|---|B
Figure S3. dsRNA/ssRNA melting curve analysis using temperature dependent IP RP HPLC analysis. A) IP RP HPLC retention times of dsRNA and ssRNA at various temperatures. B) Melting curve of ssRNA and dsRNA based on retention time plotted against temperature. The results show the clear shift in retention time of the duplex dsRNA as it melts to ssRNA (78-80 °C). In contrast no melting or large change in retention time of the ssRNA is observed from 48 °C to 80 °C under the IP RP HPLC conditions.
Supplementary Figure 3

## Slide 4
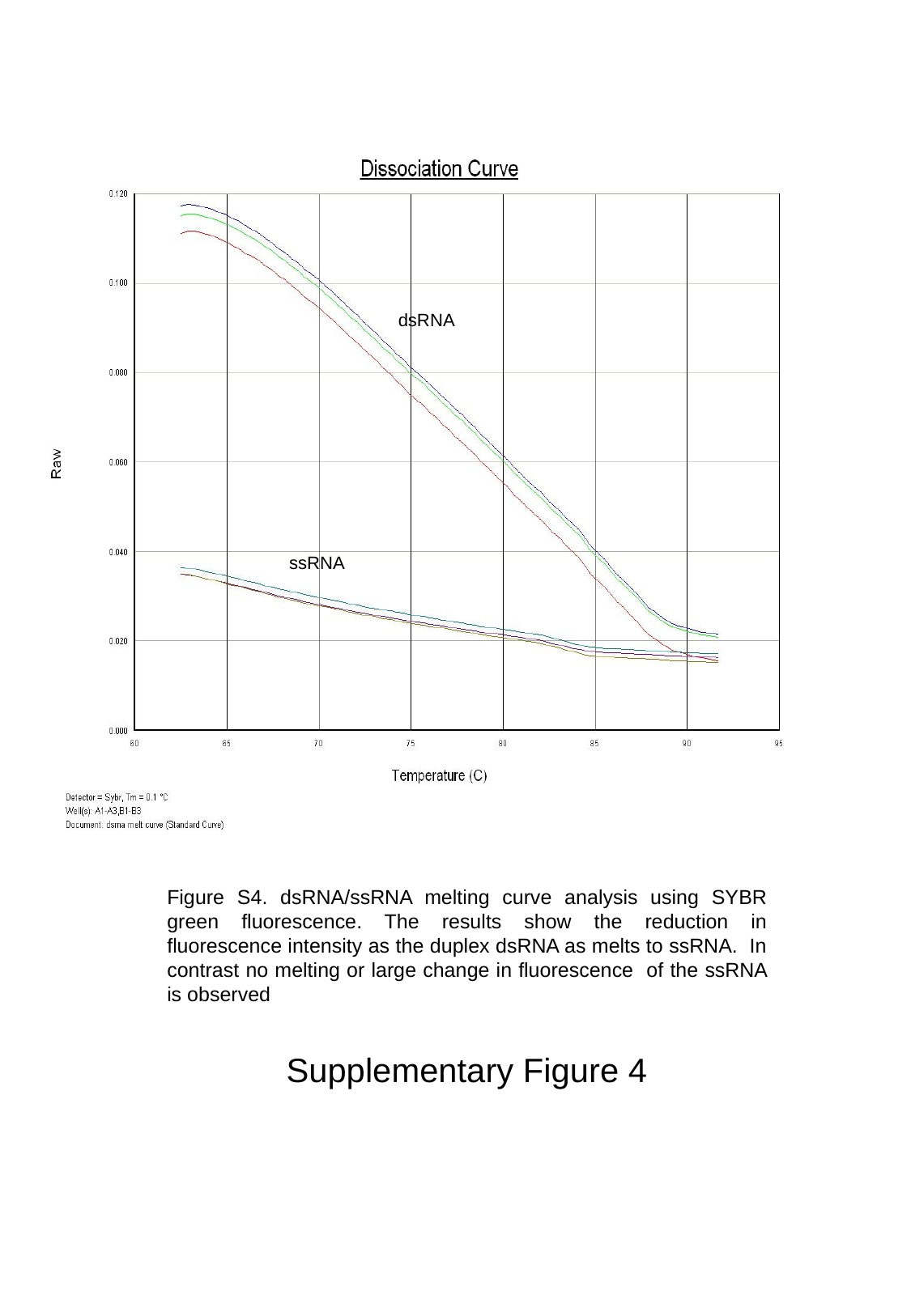

dsRNA
ssRNA
Figure S4. dsRNA/ssRNA melting curve analysis using SYBR green fluorescence. The results show the reduction in fluorescence intensity as the duplex dsRNA as melts to ssRNA. In contrast no melting or large change in fluorescence of the ssRNA is observed
Supplementary Figure 4

## Slide 5
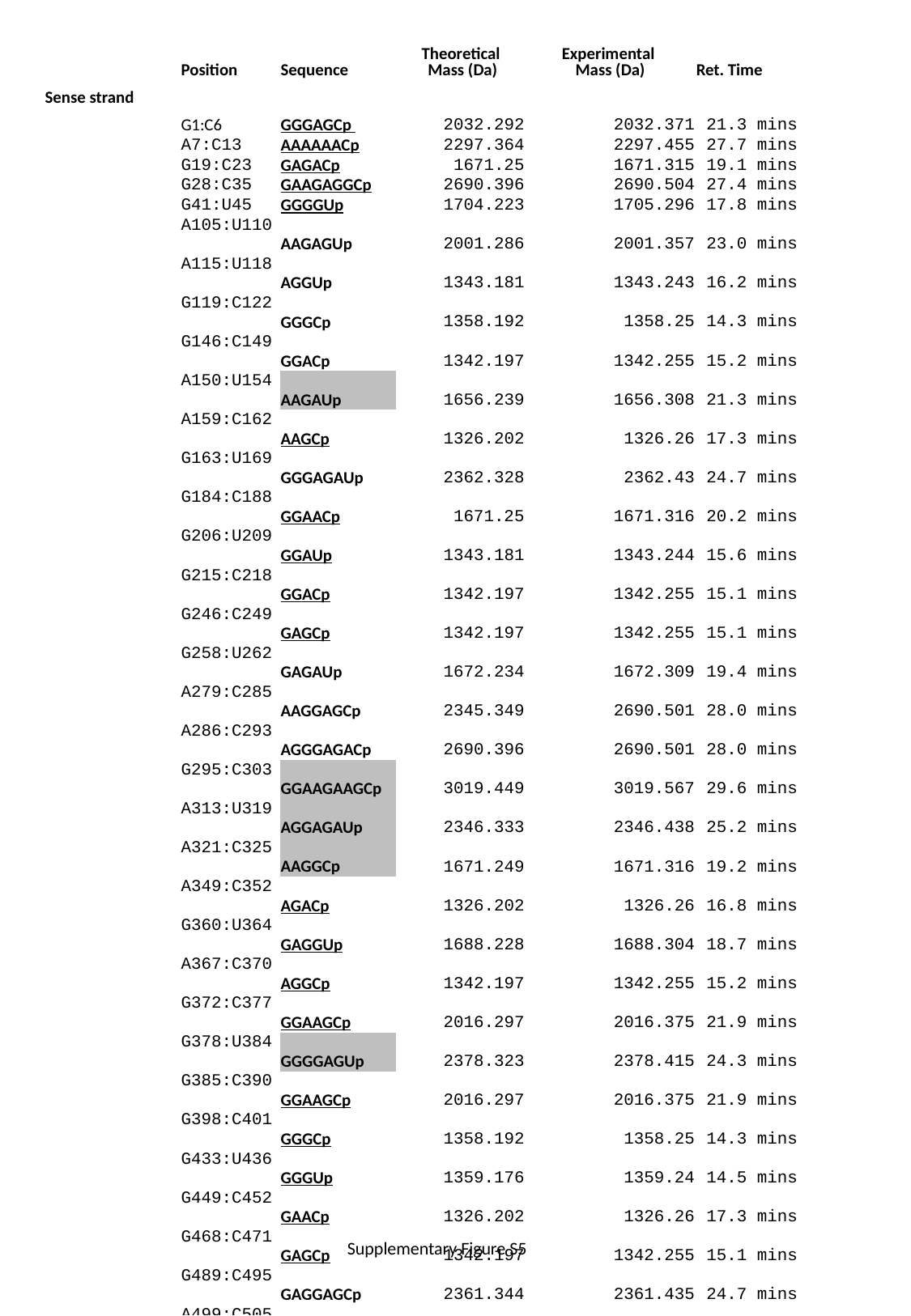

| | Position | Sequence | Theoretical Mass (Da) | Experimental Mass (Da) | Ret. Time |
| --- | --- | --- | --- | --- | --- |
| Sense strand | | | | | |
| | G1:C6 | GGGAGCp | 2032.292 | 2032.371 | 21.3 mins |
| | A7:C13 | AAAAAACp | 2297.364 | 2297.455 | 27.7 mins |
| | G19:C23 | GAGACp | 1671.25 | 1671.315 | 19.1 mins |
| | G28:C35 | GAAGAGGCp | 2690.396 | 2690.504 | 27.4 mins |
| | G41:U45 | GGGGUp | 1704.223 | 1705.296 | 17.8 mins |
| | A105:U110 | AAGAGUp | 2001.286 | 2001.357 | 23.0 mins |
| | A115:U118 | AGGUp | 1343.181 | 1343.243 | 16.2 mins |
| | G119:C122 | GGGCp | 1358.192 | 1358.25 | 14.3 mins |
| | G146:C149 | GGACp | 1342.197 | 1342.255 | 15.2 mins |
| | A150:U154 | AAGAUp | 1656.239 | 1656.308 | 21.3 mins |
| | A159:C162 | AAGCp | 1326.202 | 1326.26 | 17.3 mins |
| | G163:U169 | GGGAGAUp | 2362.328 | 2362.43 | 24.7 mins |
| | G184:C188 | GGAACp | 1671.25 | 1671.316 | 20.2 mins |
| | G206:U209 | GGAUp | 1343.181 | 1343.244 | 15.6 mins |
| | G215:C218 | GGACp | 1342.197 | 1342.255 | 15.1 mins |
| | G246:C249 | GAGCp | 1342.197 | 1342.255 | 15.1 mins |
| | G258:U262 | GAGAUp | 1672.234 | 1672.309 | 19.4 mins |
| | A279:C285 | AAGGAGCp | 2345.349 | 2690.501 | 28.0 mins |
| | A286:C293 | AGGGAGACp | 2690.396 | 2690.501 | 28.0 mins |
| | G295:C303 | GGAAGAAGCp | 3019.449 | 3019.567 | 29.6 mins |
| | A313:U319 | AGGAGAUp | 2346.333 | 2346.438 | 25.2 mins |
| | A321:C325 | AAGGCp | 1671.249 | 1671.316 | 19.2 mins |
| | A349:C352 | AGACp | 1326.202 | 1326.26 | 16.8 mins |
| | G360:U364 | GAGGUp | 1688.228 | 1688.304 | 18.7 mins |
| | A367:C370 | AGGCp | 1342.197 | 1342.255 | 15.2 mins |
| | G372:C377 | GGAAGCp | 2016.297 | 2016.375 | 21.9 mins |
| | G378:U384 | GGGGAGUp | 2378.323 | 2378.415 | 24.3 mins |
| | G385:C390 | GGAAGCp | 2016.297 | 2016.375 | 21.9 mins |
| | G398:C401 | GGGCp | 1358.192 | 1358.25 | 14.3 mins |
| | G433:U436 | GGGUp | 1359.176 | 1359.24 | 14.5 mins |
| | G449:C452 | GAACp | 1326.202 | 1326.26 | 17.3 mins |
| | G468:C471 | GAGCp | 1342.197 | 1342.255 | 15.1 mins |
| | G489:C495 | GAGGAGCp | 2361.344 | 2361.435 | 24.7 mins |
| | A499:C505 | AGAAGGCp | 2345.349 | 2345.445 | 25.2 mins |
| | G530:U535 | GGAAAUp | 2001.286 | 2001.365 | 24.2 mins |
| | G549:U552 | GGAUp | 1343.181 | 1343.244 | 15.6 mins |
| | A561:U564 | AAGUp | 1327.186 | 1326.26 | 17.4 mins |
| | G565:C569 | GGGACp | 1687.244 | 1687.312 | 18.3 mins |
| | G573:C578 | GAGAACp | 2000.302 | 2000.38 | 22.7 mins |
| | A579:U585 | AAGAAGUp | 2330.338 | 2330.431 | 27.1 mins |
| | G586:C593 | GGAAGAACp | 2674.401 | 2674.507 | 27.7 mins |
| | G596:U600 | GGAAUp | 1672.234 | 1672.307 | 19.5 mins |
| | G611:C615 | GGAGCp | 1687.244 | 1687.312 | 18.3 mins |
| | A627:C632 | AAGGGCp | 2016.297 | 2016.375 | 23.1 mins |
| | G635:C638 | GAACp | 1326.202 | 1326.26 | 17.3 mins |
| | G647:U650 | GGGUp | 1359.176 | 1359.24 | 14.5 mins |
| | A651:C655 | AAAACp | 1639.26 | 1639.326 | 21.7 mins |
| | G661:C665 | GGGGCp | 1703.239 | 1703.307 | 17.7 mins |
| | G672:U677 | GGGGGUp | 2049.27 | 2049.35 | 21.0 mins |
| | G683:U687 | GGGGUp | 1704.223 | 1704.293 | 18.0 mins |
Supplementary Figure S5

## Slide 6
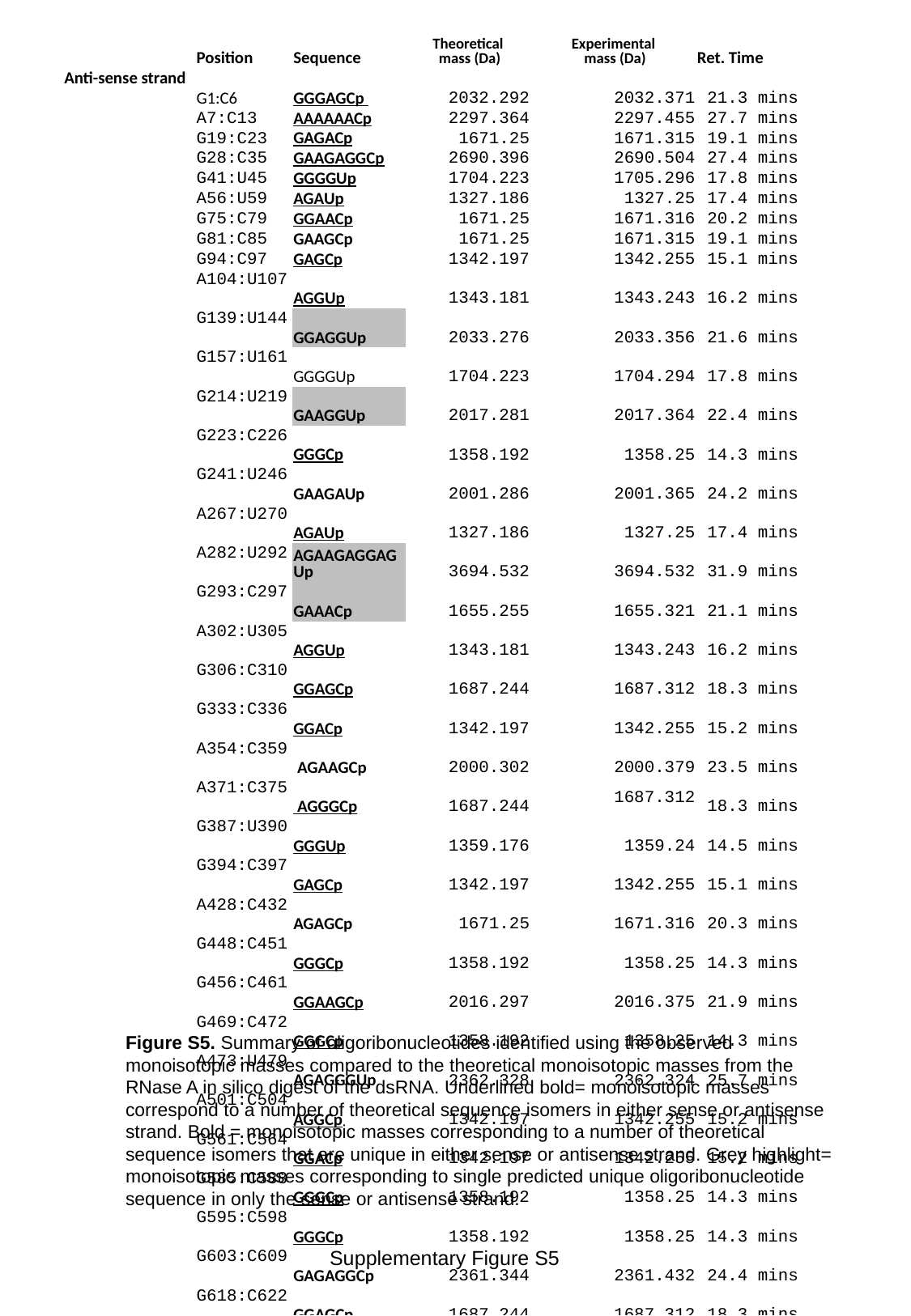

| | Position | Sequence | Theoretical mass (Da) | Experimental mass (Da) | Ret. Time |
| --- | --- | --- | --- | --- | --- |
| Anti-sense strand | | | | | |
| | G1:C6 | GGGAGCp | 2032.292 | 2032.371 | 21.3 mins |
| | A7:C13 | AAAAAACp | 2297.364 | 2297.455 | 27.7 mins |
| | G19:C23 | GAGACp | 1671.25 | 1671.315 | 19.1 mins |
| | G28:C35 | GAAGAGGCp | 2690.396 | 2690.504 | 27.4 mins |
| | G41:U45 | GGGGUp | 1704.223 | 1705.296 | 17.8 mins |
| | A56:U59 | AGAUp | 1327.186 | 1327.25 | 17.4 mins |
| | G75:C79 | GGAACp | 1671.25 | 1671.316 | 20.2 mins |
| | G81:C85 | GAAGCp | 1671.25 | 1671.315 | 19.1 mins |
| | G94:C97 | GAGCp | 1342.197 | 1342.255 | 15.1 mins |
| | A104:U107 | AGGUp | 1343.181 | 1343.243 | 16.2 mins |
| | G139:U144 | GGAGGUp | 2033.276 | 2033.356 | 21.6 mins |
| | G157:U161 | GGGGUp | 1704.223 | 1704.294 | 17.8 mins |
| | G214:U219 | GAAGGUp | 2017.281 | 2017.364 | 22.4 mins |
| | G223:C226 | GGGCp | 1358.192 | 1358.25 | 14.3 mins |
| | G241:U246 | GAAGAUp | 2001.286 | 2001.365 | 24.2 mins |
| | A267:U270 | AGAUp | 1327.186 | 1327.25 | 17.4 mins |
| | A282:U292 | AGAAGAGGAGUp | 3694.532 | 3694.532 | 31.9 mins |
| | G293:C297 | GAAACp | 1655.255 | 1655.321 | 21.1 mins |
| | A302:U305 | AGGUp | 1343.181 | 1343.243 | 16.2 mins |
| | G306:C310 | GGAGCp | 1687.244 | 1687.312 | 18.3 mins |
| | G333:C336 | GGACp | 1342.197 | 1342.255 | 15.2 mins |
| | A354:C359 | AGAAGCp | 2000.302 | 2000.379 | 23.5 mins |
| | A371:C375 | AGGGCp | 1687.244 | 1687.312 | 18.3 mins |
| | G387:U390 | GGGUp | 1359.176 | 1359.24 | 14.5 mins |
| | G394:C397 | GAGCp | 1342.197 | 1342.255 | 15.1 mins |
| | A428:C432 | AGAGCp | 1671.25 | 1671.316 | 20.3 mins |
| | G448:C451 | GGGCp | 1358.192 | 1358.25 | 14.3 mins |
| | G456:C461 | GGAAGCp | 2016.297 | 2016.375 | 21.9 mins |
| | G469:C472 | GGGCp | 1358.192 | 1358.25 | 14.3 mins |
| | A473:U479 | AGAGGGUp | 2362.328 | 2362.324 | 25.7 mins |
| | A501:C504 | AGGCp | 1342.197 | 1342.255 | 15.2 mins |
| | G561:C564 | GGACp | 1342.197 | 1342.255 | 15.2 mins |
| | G585:C588 | GGGCp | 1358.192 | 1358.25 | 14.3 mins |
| | G595:C598 | GGGCp | 1358.192 | 1358.25 | 14.3 mins |
| | G603:C609 | GAGAGGCp | 2361.344 | 2361.432 | 24.4 mins |
| | G618:C622 | GGAGCp | 1687.244 | 1687.312 | 18.3 mins |
| | G634:U637 | GAGUp | 1343.181 | 1343.243 | 16.2 mins |
| | A644:C648 | AGGGCp | 1687.244 | 1687.312 | 18.3 mins |
| | G661:C665 | GGGGCp | 1703.239 | 1703.307 | 17.7 mins |
| | G672:U677 | GGGGGUp | 2049.27 | 2049.35 | 21.0 mins |
| | G683:U687 | GGGGUp | 1704.223 | 1704.294 | 17.8 mins |
Figure S5. Summary of oligoribonucleotides identified using the observed monoisotopic masses compared to the theoretical monoisotopic masses from the RNase A in silico digest of the dsRNA. Underlined bold= monoisotopic masses correspond to a number of theoretical sequence isomers in either sense or antisense strand. Bold = monoisotopic masses corresponding to a number of theoretical sequence isomers that are unique in either sense or antisense strand. Grey highlight= monoisotopic masses corresponding to single predicted unique oligoribonucleotide sequence in only the sense or antisense strand.
Supplementary Figure S5

## Slide 7
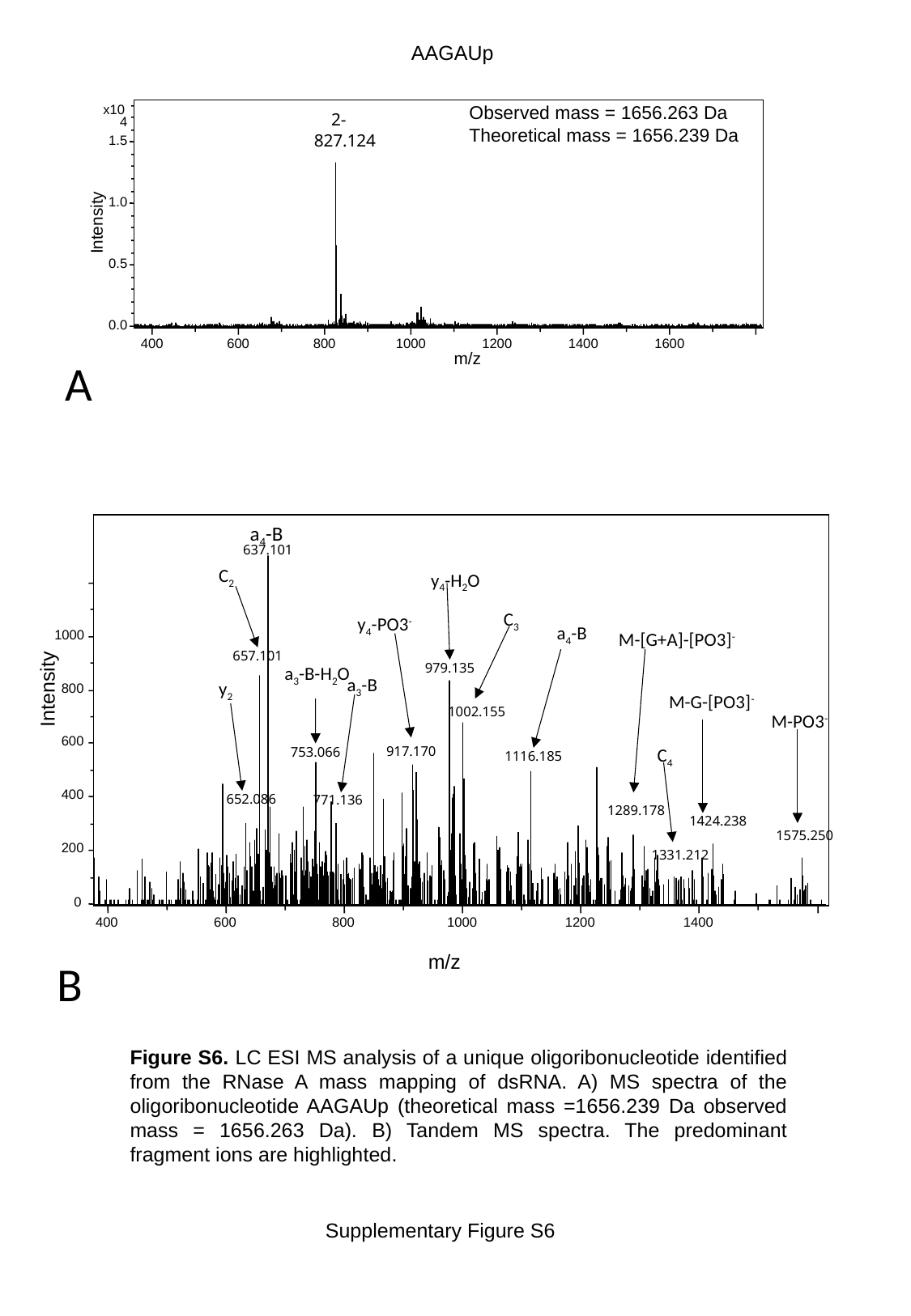

AAGAUp
x10
Observed mass = 1656.263 Da
Theoretical mass = 1656.239 Da
2-
4
827.124
1.5
Intensity
1.0
0.5
0.0
400
600
800
1000
1200
1400
1600
m/z
A
a4-B
637.101
C2
y4-H2O
C3
y4-PO3-
a4-B
M-[G+A]-[PO3]-
1000
657.101
a3-B-H2O
979.135
a3-B
y2
Intensity
800
M-G-[PO3]-
1002.155
M-PO3-
600
C4
917.170
753.066
1116.185
400
652.086
771.136
1289.178
1424.238
1575.250
200
1331.212
0
400
600
800
1000
1200
1400
m/z
B
Figure S6. LC ESI MS analysis of a unique oligoribonucleotide identified from the RNase A mass mapping of dsRNA. A) MS spectra of the oligoribonucleotide AAGAUp (theoretical mass =1656.239 Da observed mass = 1656.263 Da). B) Tandem MS spectra. The predominant fragment ions are highlighted.
Supplementary Figure S6

## Slide 8
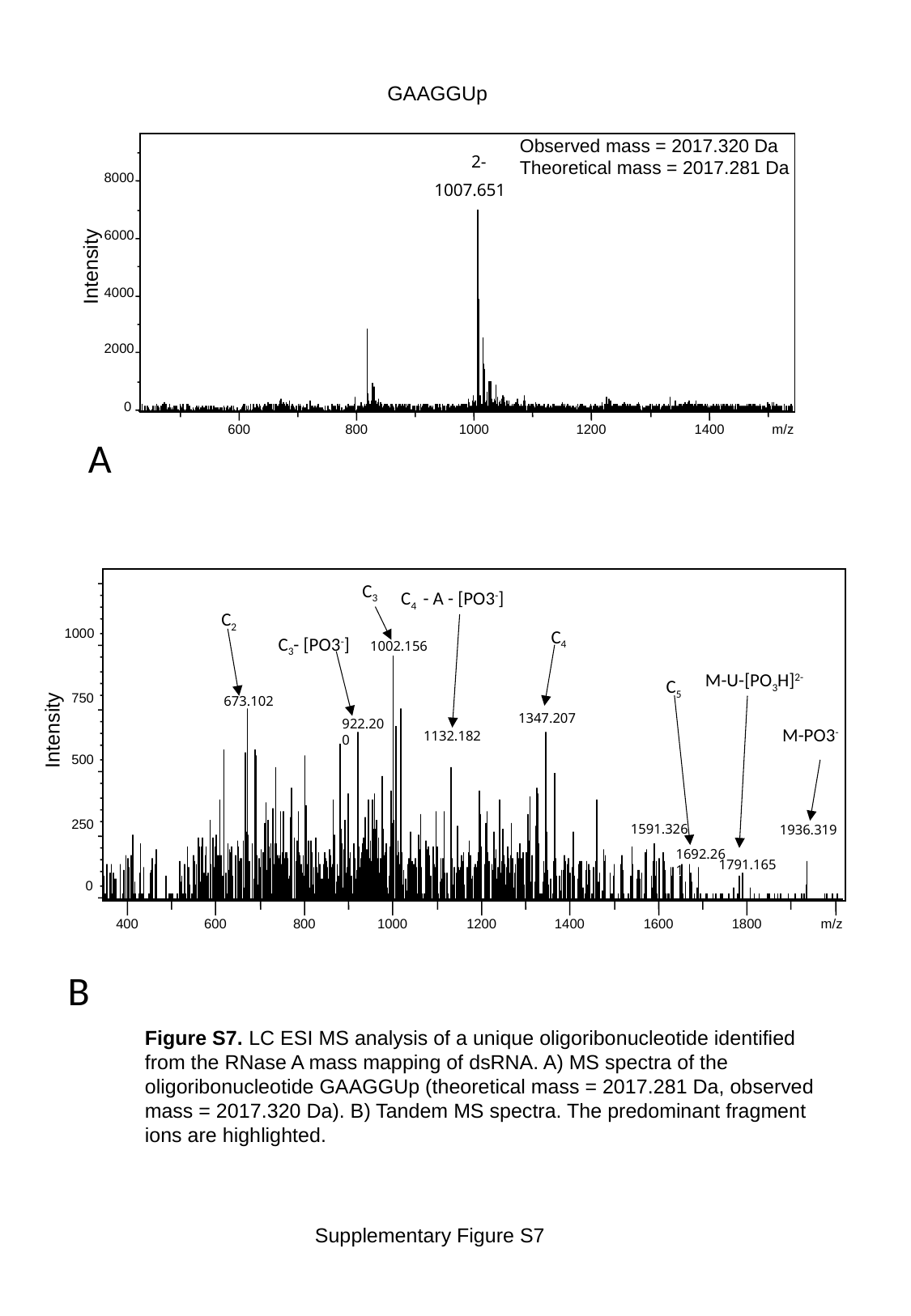

GAAGGUp
Observed mass = 2017.320 Da
Theoretical mass = 2017.281 Da
2-
8000
1007.651
6000
Intensity
4000
2000
0
600
800
1000
1200
1400
m/z
A
C3
C4 - A - [PO3-]
C2
C4
1000
C3- [PO3-]
1002.156
M-U-[PO3H]2-
C5
Intensity
750
673.102
1347.207
922.200
M-PO3-
1132.182
500
250
1591.326
1936.319
1692.261
1791.165
0
400
600
800
1000
1200
1400
1600
1800
m/z
B
Figure S7. LC ESI MS analysis of a unique oligoribonucleotide identified from the RNase A mass mapping of dsRNA. A) MS spectra of the oligoribonucleotide GAAGGUp (theoretical mass = 2017.281 Da, observed mass = 2017.320 Da). B) Tandem MS spectra. The predominant fragment ions are highlighted.
Supplementary Figure S7

## Slide 9
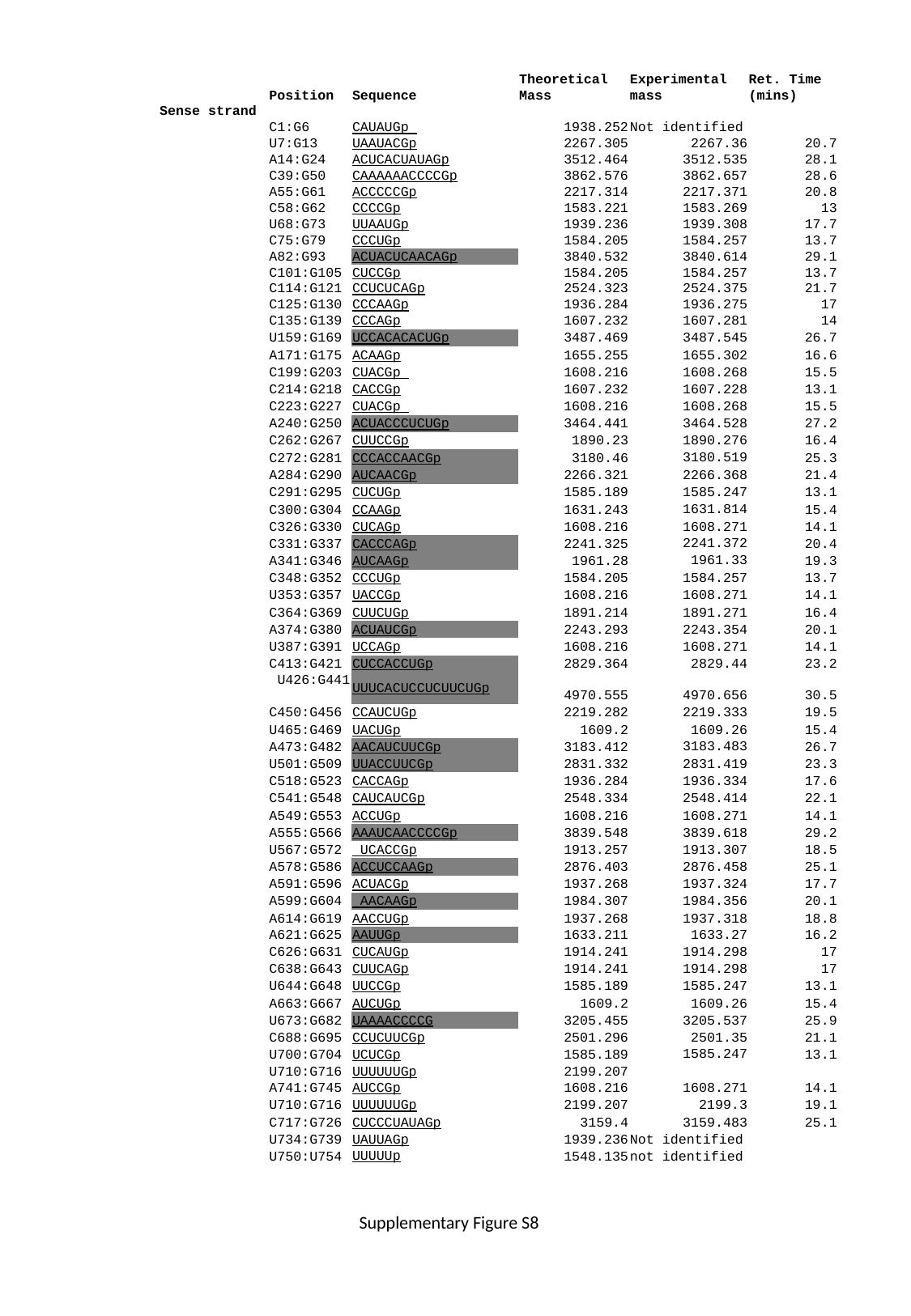

| | Position | Sequence | Theoretical Mass | Experimental mass | Ret. Time (mins) |
| --- | --- | --- | --- | --- | --- |
| Sense strand | | | | | |
| | C1:G6 | CAUAUGp | 1938.252 | Not identified | |
| | U7:G13 | UAAUACGp | 2267.305 | 2267.36 | 20.7 |
| | A14:G24 | ACUCACUAUAGp | 3512.464 | 3512.535 | 28.1 |
| | C39:G50 | CAAAAAACCCCGp | 3862.576 | 3862.657 | 28.6 |
| | A55:G61 | ACCCCCGp | 2217.314 | 2217.371 | 20.8 |
| | C58:G62 | CCCCGp | 1583.221 | 1583.269 | 13 |
| | U68:G73 | UUAAUGp | 1939.236 | 1939.308 | 17.7 |
| | C75:G79 | CCCUGp | 1584.205 | 1584.257 | 13.7 |
| | A82:G93 | ACUACUCAACAGp | 3840.532 | 3840.614 | 29.1 |
| | C101:G105 | CUCCGp | 1584.205 | 1584.257 | 13.7 |
| | C114:G121 | CCUCUCAGp | 2524.323 | 2524.375 | 21.7 |
| | C125:G130 | CCCAAGp | 1936.284 | 1936.275 | 17 |
| | C135:G139 | CCCAGp | 1607.232 | 1607.281 | 14 |
| | U159:G169 | UCCACACACUGp | 3487.469 | 3487.545 | 26.7 |
| | A171:G175 | ACAAGp | 1655.255 | 1655.302 | 16.6 |
| | C199:G203 | CUACGp | 1608.216 | 1608.268 | 15.5 |
| | C214:G218 | CACCGp | 1607.232 | 1607.228 | 13.1 |
| | C223:G227 | CUACGp | 1608.216 | 1608.268 | 15.5 |
| | A240:G250 | ACUACCCUCUGp | 3464.441 | 3464.528 | 27.2 |
| | C262:G267 | CUUCCGp | 1890.23 | 1890.276 | 16.4 |
| | C272:G281 | CCCACCAACGp | 3180.46 | 3180.519 | 25.3 |
| | A284:G290 | AUCAACGp | 2266.321 | 2266.368 | 21.4 |
| | C291:G295 | CUCUGp | 1585.189 | 1585.247 | 13.1 |
| | C300:G304 | CCAAGp | 1631.243 | 1631.814 | 15.4 |
| | C326:G330 | CUCAGp | 1608.216 | 1608.271 | 14.1 |
| | C331:G337 | CACCCAGp | 2241.325 | 2241.372 | 20.4 |
| | A341:G346 | AUCAAGp | 1961.28 | 1961.33 | 19.3 |
| | C348:G352 | CCCUGp | 1584.205 | 1584.257 | 13.7 |
| | U353:G357 | UACCGp | 1608.216 | 1608.271 | 14.1 |
| | C364:G369 | CUUCUGp | 1891.214 | 1891.271 | 16.4 |
| | A374:G380 | ACUAUCGp | 2243.293 | 2243.354 | 20.1 |
| | U387:G391 | UCCAGp | 1608.216 | 1608.271 | 14.1 |
| | C413:G421 | CUCCACCUGp | 2829.364 | 2829.44 | 23.2 |
| | U426:G441 | UUUCACUCCUCUUCUGp | 4970.555 | 4970.656 | 30.5 |
| | C450:G456 | CCAUCUGp | 2219.282 | 2219.333 | 19.5 |
| | U465:G469 | UACUGp | 1609.2 | 1609.26 | 15.4 |
| | A473:G482 | AACAUCUUCGp | 3183.412 | 3183.483 | 26.7 |
| | U501:G509 | UUACCUUCGp | 2831.332 | 2831.419 | 23.3 |
| | C518:G523 | CACCAGp | 1936.284 | 1936.334 | 17.6 |
| | C541:G548 | CAUCAUCGp | 2548.334 | 2548.414 | 22.1 |
| | A549:G553 | ACCUGp | 1608.216 | 1608.271 | 14.1 |
| | A555:G566 | AAAUCAACCCCGp | 3839.548 | 3839.618 | 29.2 |
| | U567:G572 | UCACCGp | 1913.257 | 1913.307 | 18.5 |
| | A578:G586 | ACCUCCAAGp | 2876.403 | 2876.458 | 25.1 |
| | A591:G596 | ACUACGp | 1937.268 | 1937.324 | 17.7 |
| | A599:G604 | AACAAGp | 1984.307 | 1984.356 | 20.1 |
| | A614:G619 | AACCUGp | 1937.268 | 1937.318 | 18.8 |
| | A621:G625 | AAUUGp | 1633.211 | 1633.27 | 16.2 |
| | C626:G631 | CUCAUGp | 1914.241 | 1914.298 | 17 |
| | C638:G643 | CUUCAGp | 1914.241 | 1914.298 | 17 |
| | U644:G648 | UUCCGp | 1585.189 | 1585.247 | 13.1 |
| | A663:G667 | AUCUGp | 1609.2 | 1609.26 | 15.4 |
| | U673:G682 | UAAAACCCCG | 3205.455 | 3205.537 | 25.9 |
| | C688:G695 | CCUCUUCGp | 2501.296 | 2501.35 | 21.1 |
| | U700:G704 | UCUCGp | 1585.189 | 1585.247 | 13.1 |
| | U710:G716 | UUUUUUGp | 2199.207 | | |
| | A741:G745 | AUCCGp | 1608.216 | 1608.271 | 14.1 |
| | U710:G716 | UUUUUUGp | 2199.207 | 2199.3 | 19.1 |
| | C717:G726 | CUCCCUAUAGp | 3159.4 | 3159.483 | 25.1 |
| | U734:G739 | UAUUAGp | 1939.236 | Not identified | |
| | U750:U754 | UUUUUp | 1548.135 | not identified | |
Supplementary Figure S8

## Slide 10
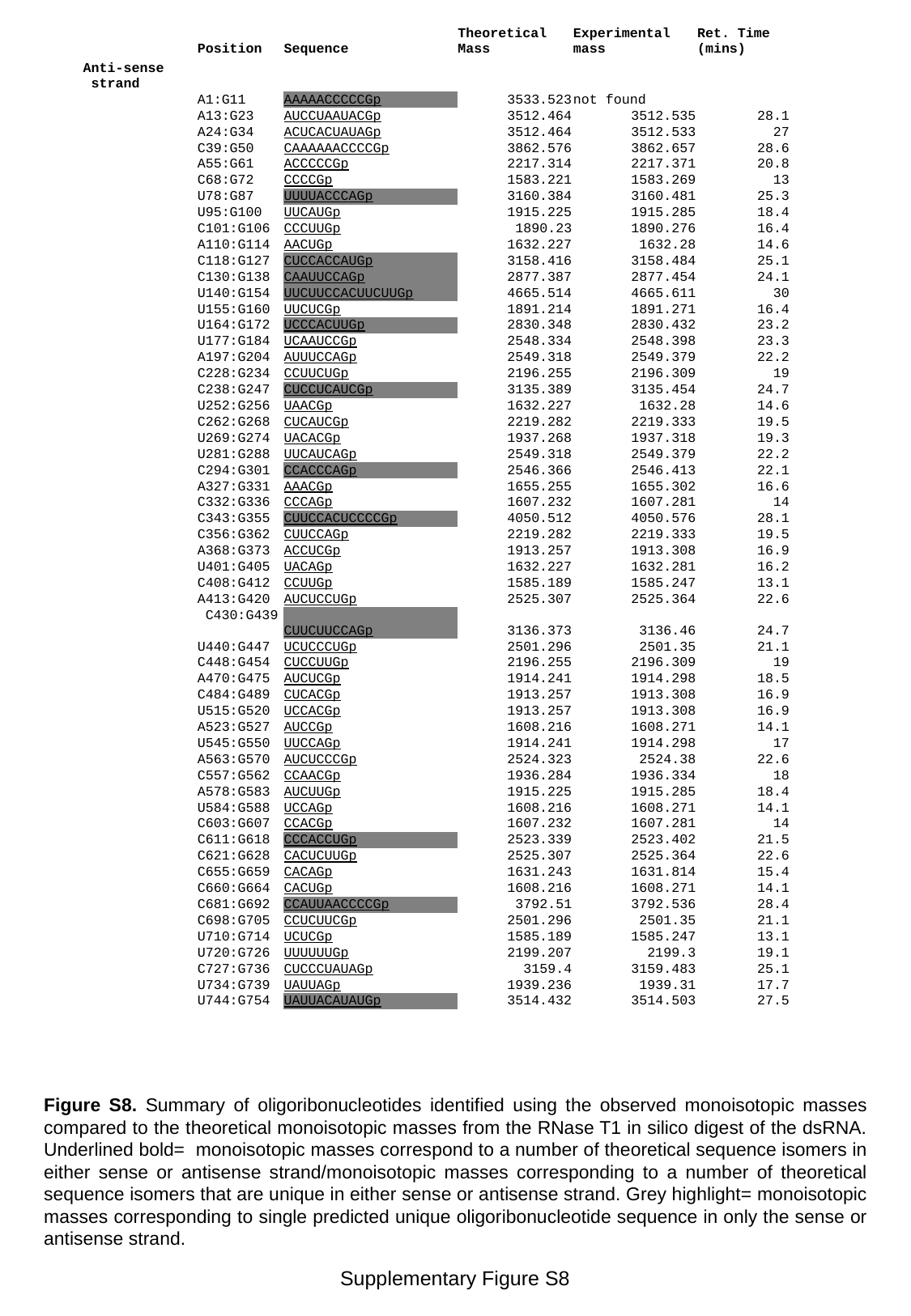

| | Position | Sequence | Theoretical Mass | Experimental mass | Ret. Time (mins) |
| --- | --- | --- | --- | --- | --- |
| Anti-sense strand | | | | | |
| | A1:G11 | AAAAACCCCCGp | 3533.523 | not found | |
| | A13:G23 | AUCCUAAUACGp | 3512.464 | 3512.535 | 28.1 |
| | A24:G34 | ACUCACUAUAGp | 3512.464 | 3512.533 | 27 |
| | C39:G50 | CAAAAAACCCCGp | 3862.576 | 3862.657 | 28.6 |
| | A55:G61 | ACCCCCGp | 2217.314 | 2217.371 | 20.8 |
| | C68:G72 | CCCCGp | 1583.221 | 1583.269 | 13 |
| | U78:G87 | UUUUACCCAGp | 3160.384 | 3160.481 | 25.3 |
| | U95:G100 | UUCAUGp | 1915.225 | 1915.285 | 18.4 |
| | C101:G106 | CCCUUGp | 1890.23 | 1890.276 | 16.4 |
| | A110:G114 | AACUGp | 1632.227 | 1632.28 | 14.6 |
| | C118:G127 | CUCCACCAUGp | 3158.416 | 3158.484 | 25.1 |
| | C130:G138 | CAAUUCCAGp | 2877.387 | 2877.454 | 24.1 |
| | U140:G154 | UUCUUCCACUUCUUGp | 4665.514 | 4665.611 | 30 |
| | U155:G160 | UUCUCGp | 1891.214 | 1891.271 | 16.4 |
| | U164:G172 | UCCCACUUGp | 2830.348 | 2830.432 | 23.2 |
| | U177:G184 | UCAAUCCGp | 2548.334 | 2548.398 | 23.3 |
| | A197:G204 | AUUUCCAGp | 2549.318 | 2549.379 | 22.2 |
| | C228:G234 | CCUUCUGp | 2196.255 | 2196.309 | 19 |
| | C238:G247 | CUCCUCAUCGp | 3135.389 | 3135.454 | 24.7 |
| | U252:G256 | UAACGp | 1632.227 | 1632.28 | 14.6 |
| | C262:G268 | CUCAUCGp | 2219.282 | 2219.333 | 19.5 |
| | U269:G274 | UACACGp | 1937.268 | 1937.318 | 19.3 |
| | U281:G288 | UUCAUCAGp | 2549.318 | 2549.379 | 22.2 |
| | C294:G301 | CCACCCAGp | 2546.366 | 2546.413 | 22.1 |
| | A327:G331 | AAACGp | 1655.255 | 1655.302 | 16.6 |
| | C332:G336 | CCCAGp | 1607.232 | 1607.281 | 14 |
| | C343:G355 | CUUCCACUCCCCGp | 4050.512 | 4050.576 | 28.1 |
| | C356:G362 | CUUCCAGp | 2219.282 | 2219.333 | 19.5 |
| | A368:G373 | ACCUCGp | 1913.257 | 1913.308 | 16.9 |
| | U401:G405 | UACAGp | 1632.227 | 1632.281 | 16.2 |
| | C408:G412 | CCUUGp | 1585.189 | 1585.247 | 13.1 |
| | A413:G420 | AUCUCCUGp | 2525.307 | 2525.364 | 22.6 |
| | C430:G439 | CUUCUUCCAGp | 3136.373 | 3136.46 | 24.7 |
| | U440:G447 | UCUCCCUGp | 2501.296 | 2501.35 | 21.1 |
| | C448:G454 | CUCCUUGp | 2196.255 | 2196.309 | 19 |
| | A470:G475 | AUCUCGp | 1914.241 | 1914.298 | 18.5 |
| | C484:G489 | CUCACGp | 1913.257 | 1913.308 | 16.9 |
| | U515:G520 | UCCACGp | 1913.257 | 1913.308 | 16.9 |
| | A523:G527 | AUCCGp | 1608.216 | 1608.271 | 14.1 |
| | U545:G550 | UUCCAGp | 1914.241 | 1914.298 | 17 |
| | A563:G570 | AUCUCCCGp | 2524.323 | 2524.38 | 22.6 |
| | C557:G562 | CCAACGp | 1936.284 | 1936.334 | 18 |
| | A578:G583 | AUCUUGp | 1915.225 | 1915.285 | 18.4 |
| | U584:G588 | UCCAGp | 1608.216 | 1608.271 | 14.1 |
| | C603:G607 | CCACGp | 1607.232 | 1607.281 | 14 |
| | C611:G618 | CCCACCUGp | 2523.339 | 2523.402 | 21.5 |
| | C621:G628 | CACUCUUGp | 2525.307 | 2525.364 | 22.6 |
| | C655:G659 | CACAGp | 1631.243 | 1631.814 | 15.4 |
| | C660:G664 | CACUGp | 1608.216 | 1608.271 | 14.1 |
| | C681:G692 | CCAUUAACCCCGp | 3792.51 | 3792.536 | 28.4 |
| | C698:G705 | CCUCUUCGp | 2501.296 | 2501.35 | 21.1 |
| | U710:G714 | UCUCGp | 1585.189 | 1585.247 | 13.1 |
| | U720:G726 | UUUUUUGp | 2199.207 | 2199.3 | 19.1 |
| | C727:G736 | CUCCCUAUAGp | 3159.4 | 3159.483 | 25.1 |
| | U734:G739 | UAUUAGp | 1939.236 | 1939.31 | 17.7 |
| | U744:G754 | UAUUACAUAUGp | 3514.432 | 3514.503 | 27.5 |
Figure S8. Summary of oligoribonucleotides identified using the observed monoisotopic masses compared to the theoretical monoisotopic masses from the RNase T1 in silico digest of the dsRNA. Underlined bold= monoisotopic masses correspond to a number of theoretical sequence isomers in either sense or antisense strand/monoisotopic masses corresponding to a number of theoretical sequence isomers that are unique in either sense or antisense strand. Grey highlight= monoisotopic masses corresponding to single predicted unique oligoribonucleotide sequence in only the sense or antisense strand.
Supplementary Figure S8
